# An Overview of Two Old Friends Associated with Platelet Redox Signaling, the Protein Disulfide Isomerase and NADPH Oxidase

**DOI:** 10.3390/biom13050848

**Published:** 2023-05-17

**Authors:** Andrés Trostchansky, Marcelo Alarcon

**Affiliations:** 1 Departamento de Bioquímica and Center for Free Radical and Biomedical Research, Facultad de Medicina, Universidad de la República, Montevideo 11800, Uruguay; 2 Thrombosis Research Center, Universidad de Talca, Talca 3460000, Chile; 3 Department of Clinical Biochemistry and Immunohematology, Faculty of Health Sciences, Universidad de Talca, Talca 3460000, Chile

**Keywords:** Protein Disulphide Isomerase, NADPH oxidase, platelets, oxidative stress, reactive oxygen species and cardiovascular diseases

## Abstract

Oxidative stress participates at the baseline of different non-communicable pathologies such as cardiovascular diseases. Excessive formation of reactive oxygen species (ROS), above the signaling levels necessary for the correct function of organelles and cells, may contribute to the non-desired effects of oxidative stress. Platelets play a relevant role in arterial thrombosis, by aggregation triggered by different agonists, where excessive ROS formation induces mitochondrial dysfunction and stimulate platelet activation and aggregation. Platelet is both a source and a target of ROS, thus we aim to analyze both the platelet enzymes responsible for ROS generation and their involvement in intracellular signal transduction pathways. Among the proteins involved in these processes are Protein Disulphide Isomerase (PDI) and NADPH oxidase (NOX) isoforms. By using bioinformatic tools and information from available databases, a complete bioinformatic analysis of the role and interactions of PDI and NOX in platelets, as well as the signal transduction pathways involved in their effects was performed. We focused the study on analyzing whether these proteins collaborate to control platelet function. The data presented in the current manuscript support the role that PDI and NOX play on activation pathways necessary for platelet activation and aggregation, as well as on the platelet signaling imbalance produced by ROS production. Our data could be used to design specific enzyme inhibitors or a dual inhibition for these enzymes with an antiplatelet effect to design promising treatments for diseases involving platelet dysfunction.

## 1. Introduction

Oxidative stress has been associated with many pathologies such as cancer, diabetes mellitus, and cardiovascular diseases due to an increase in the production of reactive oxygen species (ROS) [1,2]. It is important to point out that in all these pathologies, the pro-oxidative environment increases the probability of suffering thrombotic events due to platelet hyperactivation; in turn, activated platelets are a great source of ROS [3,4]. Therefore, the platelet is both a source and a target of ROS, so it is relevant to study both the platelets’ enzymes responsible for ROS generation and their involvement in intracellular signal transduction pathways [3,5,6]. Two critical enzymes participate in the regulation of intraplatelet ROS levels: Protein Disulfide Isomerase (PDI) and NADPH Oxidase (NOX) [7].

PDI is expressed in almost all mammalian tissues and is detected in the endoplasmic reticulum, nucleus, cytosol, cell surface, and extracellular space [8], playing an essential role in cell functions. PDI is a member of a large family of dithiol/disulfide oxidoreductases (thioredoxin superfamily) which catalyzes the oxidation, reduction, and isomerization of disulfide bonds [9]. For the latter, the catalytic vicinal active site thiols (-CXXC-) at the active sites are necessary to exert enzymatic activity [10]. There are 20 known members of the thiol isomerase family, of which at the vascular level the most important are seven (PDI (P4HB), ERp57 (PDIA3), ERp72 (PDIA4), ERp5 (PDIA6), ERp29 (ERP29), ERp44 (ERP44), and TMX3 (TMX3)) [11].

Platelets contain four members of the PDI family (P4HB, PDIA3, PDIA4, and PDIA6). These isoforms have been identified on the surface of resting platelets, or secreted and recruited to the platelet surface in response to platelet activation [11,12]. P4HB, PDIA3, and PDIA6 were also found to regulate platelet function and thrombus formation in vivo [11,12]. Importantly, it has been reported that PDI regulates the production of ROS by aiding the correct assembly of NOX [11]. Nevertheless, the specific roles of these PDI have not been thoroughly explored.

NOX is the major contributor of ROS in cells; either superoxide (O_2_^.−^) or its dismutation product hydrogen peroxide (H_2_O_2_) can act as second messengers when produced in excess can cause oxidative stress and cellular dysfunction [8]. The NOX family comprises seven different isoforms (NOX1, NOX2, NOX3, NOX4, NOX5, DUOX1, and DUOX2). Superoxide can reduce the bioavailability of nitric oxide (^.^NO) and serve as a precursor for other species such as H_2_O_2_ and peroxynitrite [13].

Platelets’ main NOX isoforms (NOX1, and NOX2) significantly contribute to ROS production and are considered responsible for platelet activation [14]. Both isoforms are inactive in resting platelets and are activated by different platelets’ agonists, forming an enzymatic complex with several cytoplasmic proteins [15].

The current manuscript aims to perform a complete bioinformatic analysis of the role and interactions of PDI and NOX in platelets, as well as the signal transduction pathways involved in their effects. In addition, we sought to examine whether these proteins collaborate to control platelet function. These data could be of great relevance for designing specific enzyme inhibitors or dual inhibition of both enzymes with an antiplatelet effect to design promising treatments for diseases involving platelet dysfunction.

## 2. Methods

### 2.1. Bioinformatic Analysis

#### 2.1.1. Candidate Targets Collection

For the bioinformatic analysis, and considering the data reported in the literature [8,16,17,18,19,20,21], the analysis was performed using P4HB, PDIA3, PDIA6, NOX1, and NOX2 for evaluating Platelet Redox Signaling.

To detect the possible candidate targets, we entered each of the enzymes into four databases (BioGRID [22], IID [23], APID [24], IntAct [25]), and selected the most over-represented targets. These databases are a biomedical interaction repository that provides a comprehensive collection of protein interactomes based on the integration of known experimentally validated protein–protein physical interactions.

For platelet target proteins, we used PlateletWeb [26] which is a platelet-related database that is also a biomedical tool for platelet protein interaction analysis in the context of integrated networks.

The targets associated with each enzyme were obtained using an online Venn diagram tool.

#### 2.1.2. Protein–Protein Interactions (PPIs) Network Construction

All selected platelet candidate targets were then searched for in the APID database [24] as this database provides a vast collection of interactomes based on experimental data. The whole database includes 90,379 distinct proteins and 678,441 singular interactions.

To further analyze the results from all target and platelet proteins targets obtained from this database, Cytoscape 3.7.2 was used as a tool to visualize and analyze the PPIs [27].

#### 2.1.3. Biological Function and Pathway Enrichment

To perform the Gene Ontology (GO) analysis and Kyoto Encyclopedia of Genes and Genomes (KEGG) pathway analysis enrichment, we used the SRplot [28] and MonaGO [29]) databases. The data from these databases allowed us to functionally characterize all genes associated with the biological processes (BP), cellular component (CC), and molecular functions (MF) as well as being able to determine the possible pathways or signal transduction pathways in which the genes could be involved.

All genes were ranked by their *p*-value, and the top ten biological processes and more relevant KEGG pathways (*p* < 0.05) were plotted.

## 3. Results

### 3.1. Screening of Candidate Targets of Protein Disulfide-Isomerase

#### 3.1.1. Interactional Network Analysis of Targets

**Figure 1:**
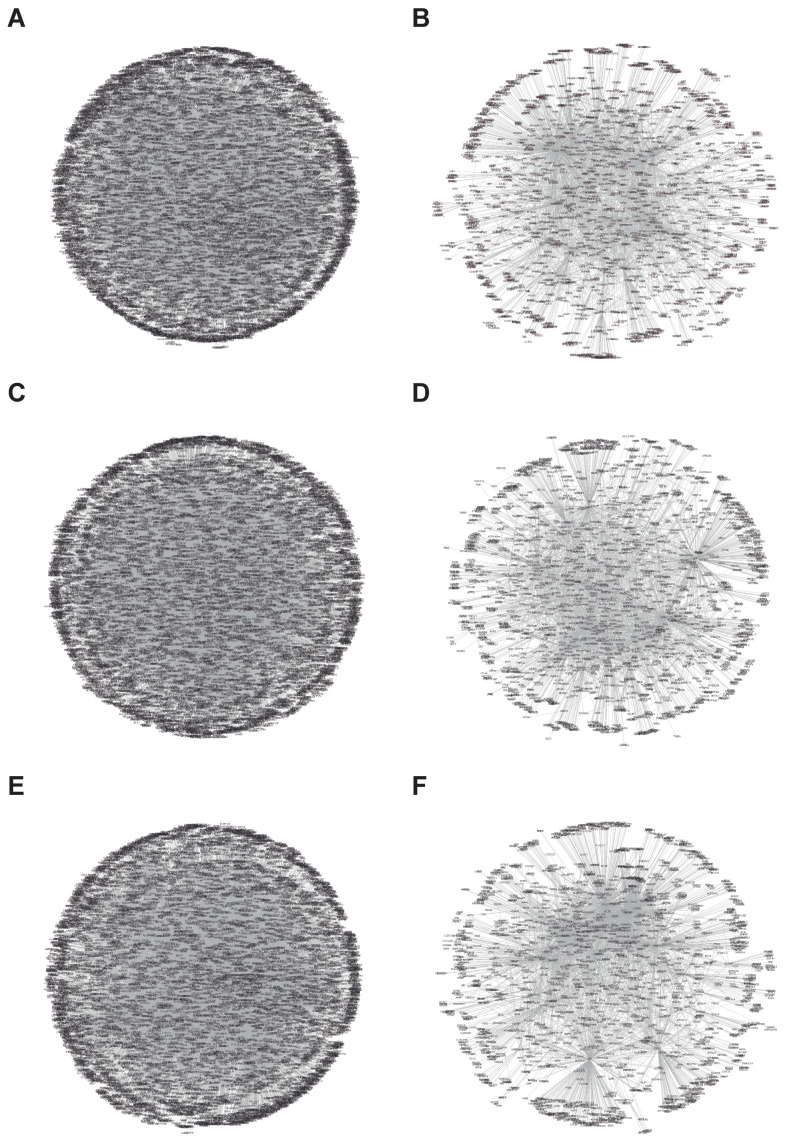
**Full protein association networks of protein disulfide-isomerase isoforms.** The network was generated with all protein interactions as explained in the Method section, and Cytoscape V3.4 (spring-embedded layout) was used. Each protein was represented by circles (nodes), and the lines (edges) connecting the two circles are indicative of an interaction between two proteins. (**A**,**B**) P4HB interactions (**A**, full protein data and **B** platelet proteins), (**C**,**D**) PDIA3 interactions (**C**, full protein data and **D** platelet proteins), and (**E**,**F**) PDIA6 interactions (**E**, full protein data and **F** platelet proteins).

We started our work by determining the fundamental importance of the platelet redox signaling of P4HB, PDIA3, and PDIA6 and how they influence platelet function. First, we constructed a network of the interactions for each PDI isoform at either all protein interaction levels (Figure 1A,C,E) or those found only in platelets (Figure 1B,D,F). It is important to point out that these networks are mathematical representations of the physical contacts between proteins in the cell. These contacts are specific, occurring between defined binding regions in the proteins. Using the Cytoscape software (spring-embedded layout), we obtained an interaction network (protein–protein) with PHB4 being the one with more interactions. The resulting network contains 8185 nodes and 20,975 edges for all proteins (Figure 1A), and 1713 nodes and 3842 edges for all proteins found in platelets (Figure 1B). It is important to note that among the proteins that present a greater number of interactions are ESR1, CUL3, NEK4, and APP. These proteins are related to oxidative stress, signal transduction, cellular communication, synaptogenesis, and synaptic plasticity. Therefore, Figure 1 shows us the great importance of the PDI isoforms in the regulation of different processes at the human being and platelet levels.

From the data in Figure 1, the detailed interactions of PDI and NOX members are reported in Table 1.

#### 3.1.2. Analysis of Biological Processes Targets

**Figure 2:**
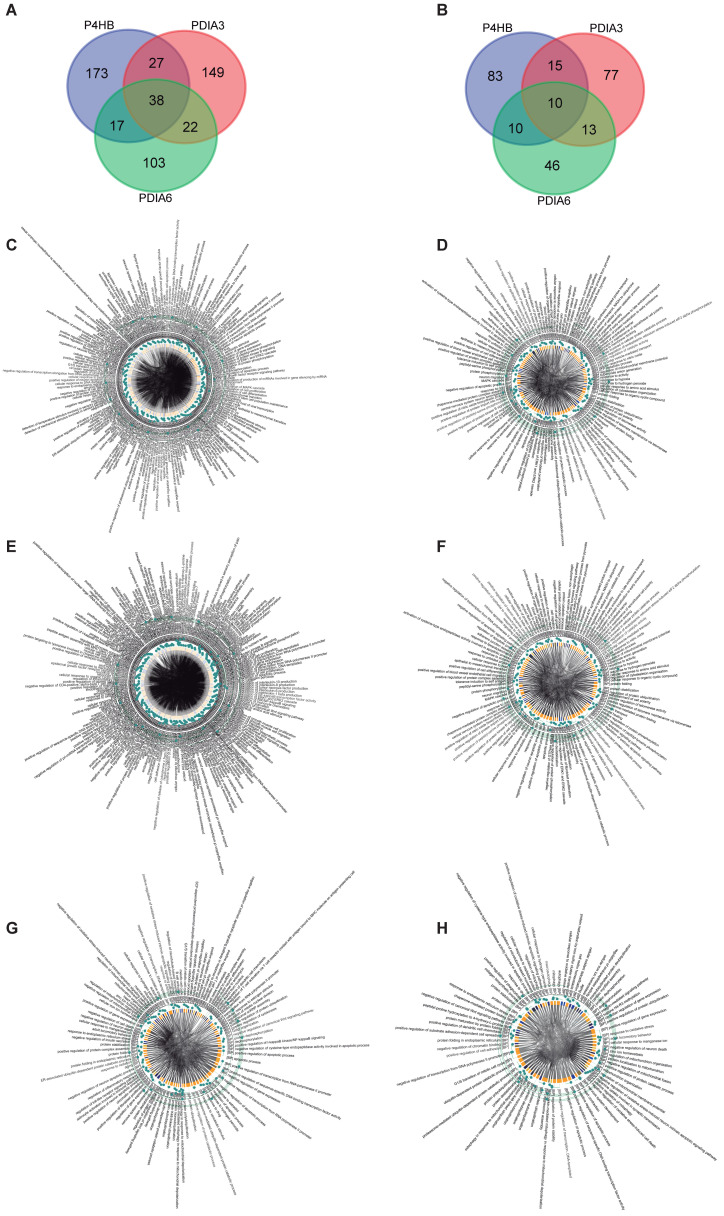
**Biological processes analysis of protein disulfide-isomerase.** (**A**) Venn diagram of full protein interactions and (**B**) platelet–protein interactions. Both diagrams display 38 and 10 overlapping genes, respectively, between PH4HB, PDIA3, and PDIA6-related target proteins. Panels (**C**–**H**) show the ChordPlot representing the connections between several biological processes. (**C**,**D**) P4HB interactions, (**E**,**F**) PDIA3 interactions, and (**G**,**H**) PDIA6 interactions.

We then wanted to determine the importance of each protein regulated by PDI. As shown in the intersection of the Venn diagram, we identified 38 and 10 common protein targets for all platelet proteins (Figure 2A,B). Some relevant platelet proteins include SOD1, UBE2M, and AAR2.

**Figure 3:**
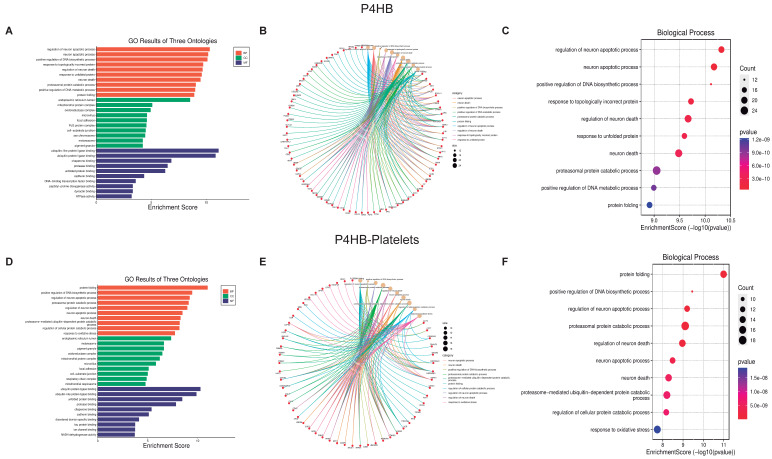
**Biological function assays of PH4HB isomerase.** Graphs associated with PH4HB isomerase were constructed with full proteins (**A**–**C**) and those associated only with platelets (**D**–**F**). (**A**,**D**) Top ten Biological processes enrichment assay of GO in terms of potential targets. (**B**,**E**) Cnetplot of GO analysis, and (**C**,**F**) Bubble chart showing the top 10 biological processes of GO terms.

**Figure 4:**
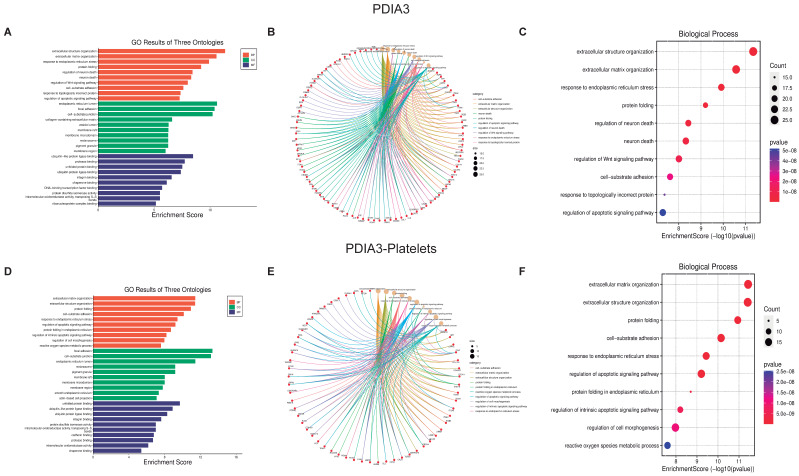
**Biological function assays of PDAI3 isomerase.** Graphs associated with PDAI3 isomerase were constructed with full proteins (**A**–**C**) and those only associated with platelets (**D**–**F**). (**A**,**D**) Top ten Biological processes enrichment assay of GO in terms of potential targets. (**B**,**E**) Cnetplot of GO analysis, and (**C**,**F**) Bubble chart showing the top 10 biological processes of GO terms.

**Figure 5:**
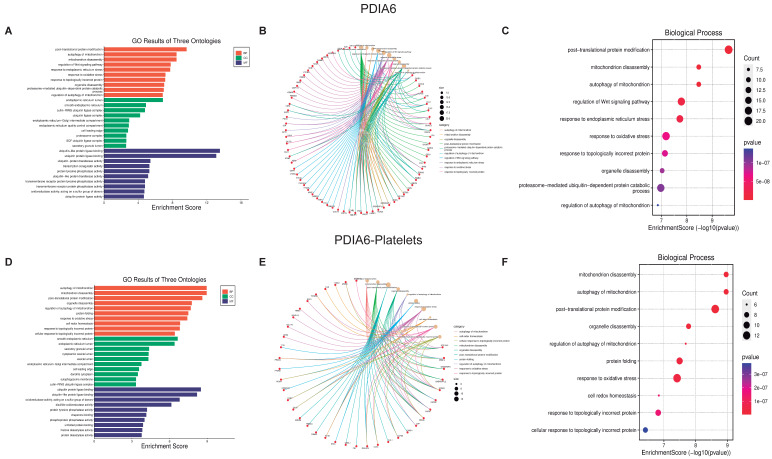
**Biological function assays of PDAI6 isomerase.** Graphs associated with PDAI6 isomerase were constructed with full proteins (**A**–**C**) and those only associated with platelets (**D**–**F**). (**A**,**D**) Top ten Biological processes enrichment assay of GO in terms of potential targets. (**B**,**E**) Cnetplot of GO analysis, and (**C**,**F**) Bubble chart showing the top 10 biological processes of GO terms.

Using GO, we constructed a Chord Plot (Figure 3, Figure 4 and Figure 5) and identified the biological processes related to the proteins that are regulated by the isomerases (Table 2). The graph represents connections between several biological processes (nodes). In the Chord Plot [30], each connection is represented by a section on the outer part of the circular layout while arcs are drawn between each biological process; the plot shows the periphery layout of the biological processes evaluated as well as the connections between them as shown in the center of the plot. The size of each arc is proportional to the importance of the network. In summary, the relevance of a protein for our organism can be determined. PDIA3 affects around 309 biological processes in our body, 207 of them in the platelet.

#### 3.1.3. Biological Function Enrichment

Now using the graphics GO Three Ontologies, Cnetplot, and Biological Processes Enrichment Score dot-plot, we analyzed the ten most essential processes regulated by these PDI isoforms and their respective interactions (Figure 3, Figure 4 and Figure 5, panels C and F). For the three PDI members, the most important processes involved in platelet function are Protein folding, Apoptotic process, and Oxidative stress. All analyzes were performed at both the cellular components (Supplementary Figures S1, S3 and S5), and molecular function (Supplementary Figures S2, S4 and S6).

The Cnetplot [31] shows the relationship between different proteins and biological processes as an entire network. Important data are obtained from the analysis of both platelets and isomerase isoforms; indeed, the data strongly support the existence of a close relationship between the regulated proteins and the different cell biological processes. Together, PDI isoforms regulate more than 200 proteins, which in turn regulate more than 2400 platelet proteins vital for signaling pathways involved in platelet function and faith. The most relevant processes regulated by P4HB and PDIA3 in platelets include, apart from cell structure organization, protein folding, ER stress, and the activation of the apoptotic pathways (panel F Figure 3, Figure 4 and Figure 5). As expected, since PDI regulates oxidant species formation in different cell types [32,33], the formation of ROS is also regulated by PDI activity [34]. ROS formation is important in regulating platelet function and aggregant capacity. PDIA6 regulates redox hemostasis, platelet response to oxidative stress, and how the platelets deal with the presence of incorrectly folded proteins (Figure 4F). Since PDI participates in the process of protein folding and the correct activation of integrins at the platelet membrane, our results confirm the importance of PDI on platelet function.

### 3.2. Screening of Candidate Targets for Protein NADPH Oxidases

#### 3.2.1. Interactional Network Analysis of Targets

**Figure 6:**
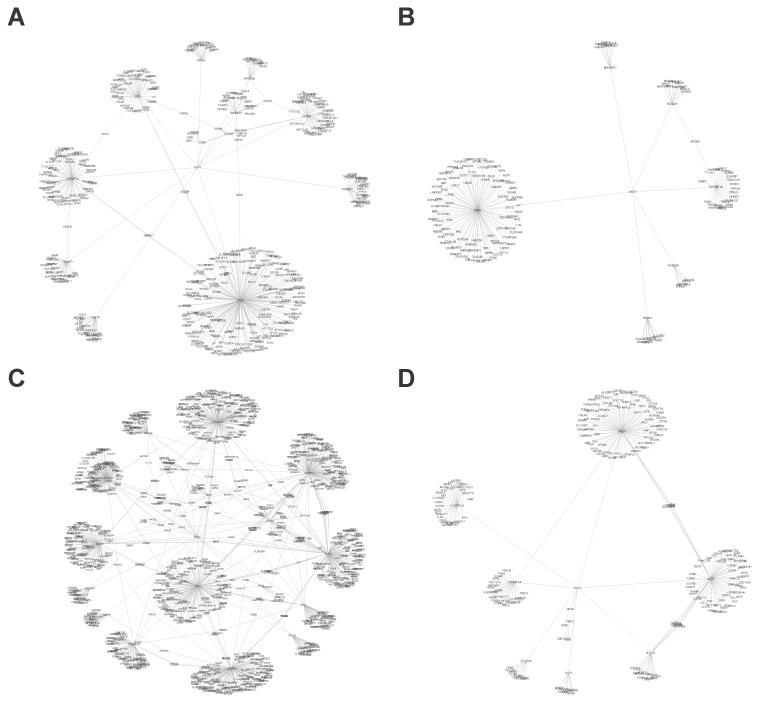
**Full protein association networks of NADPH Oxidase isoforms.** The network was generated with the full protein interactions, and Cytoscape V3.4 (spring-embedded layout) was used. Each protein was represented by circles (nodes), and the lines (edges) connecting the two circles reference an interaction between two proteins. (**A**,**B**) NOX1 interactions (**A**, full proteins and **B** platelet proteins), and (**C**,**D**) NOX2 interactions (**C**, full proteins and **D** platelet proteins).

One of the most relevant enzymatic complexes that require PDI for its activity is NOX [33]. NOX is an enzymatic complex that produces O_2_^.−^ in several cell types, platelets, and macrophages. Superoxide then dismutates to H_2_O_2_ which can decrease the reduction potential of the cell, i.e., decreasing reduced glutathione levels, or activating cell signaling pathways [35]. Platelets’ main isoforms of the NOX family are NOX1 and NOX2 [36]. It has been recently reported that depending on the agonist of platelet activation, the isoform of NOX generates O_2_^.−^; while collagen mainly induces ROS formation in platelets through NOX1, thrombin leads to NOX2 activation and platelet ROS generation. As previously performed for the PDI family, we analyzed the interactions between proteins for the two NOX isoforms (Figure 6, all protein interactions (left) and the platelet interactions (right)), being NOX2 the protein with the highest amount of interactions (Table 1). In the analysis of the obtained network, we found that it contained 1181 nodes and 1349 edges (Figure 6C) for all protein interactions, 242 nodes, and 244 edges in platelets (Figure 6D). As with PDI isoforms, we conclude that NOX can interact with and control an important number of proteins. It is important to highlight that 571 proteins are regulated by both proteins (PDI and NOX), including RAC1, KRAS, CUL1, CUL2, and CUL3.

**Figure 7:**
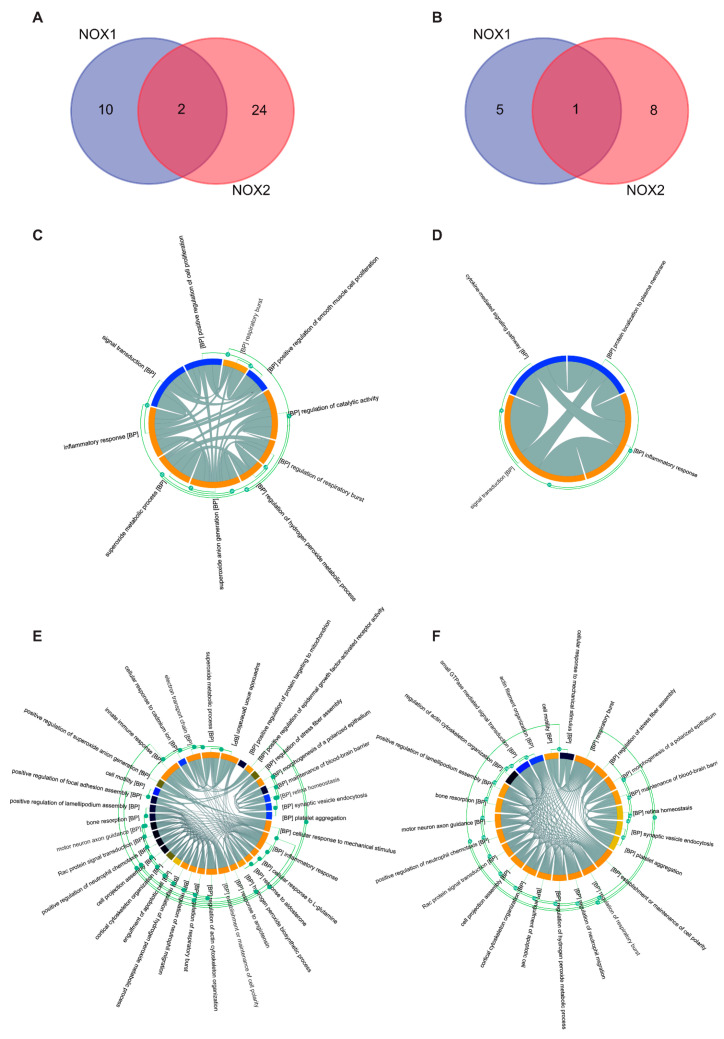
**Biological Processes analysis of NADPH Oxidases.** (**A**) Venn diagram for all protein interactions and (**B**) platelet protein interactions. Both diagrams display 2 and 1 overlapping genes, respectively, between NOX1 and NOX2-related target proteins. (**C**–**F**) show the ChordPlot represents the connections between several biological processes, (**C**,**D**) NOX1 interactions, (**E**,**F**) NOX2 interactions.

As shown in the intersection by the Venn diagram, when proteins regulated by NOX isoforms were studied, only two and one common proteins were identified for all and platelet proteins, respectively (Figure 7A,B). The protein present in platelets is tumor necrosis factor receptor 1 (TNFRSF1A), which has been reported to be crucial for platelet activation [37,38]. Using Chorplot we found that NOX affects more than 40 biological processes in the body and about 27 in the platelet (Table 2). Among these processes, relevant biological actions are affected by both NOX isoforms, and some of them were also regulated by PDI. It is worth noting that thiol-dependent signaling pathways, respiratory bursts, and inflammatory responses were regulated by either NOX or PDI. Overall, our results support the great relevance of these proteins in platelet function.

#### 3.2.2. Biological Function of NOX Isoforms

**Figure 8:**
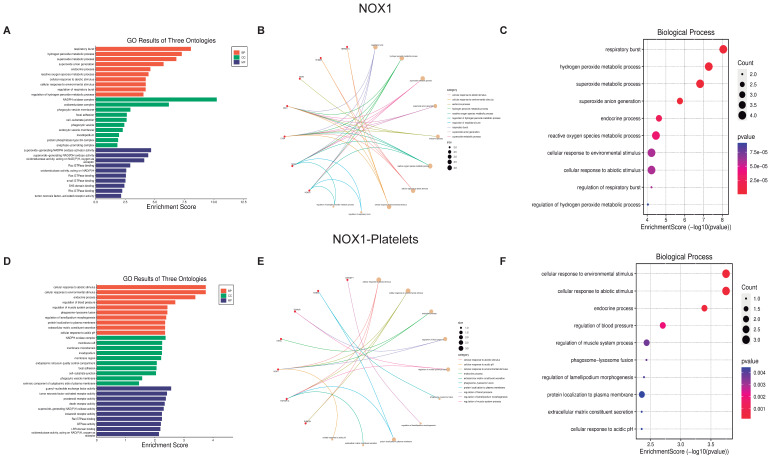
**Biological function assays of NOX1.** The graphs associated with NOX1 were constructed with all the proteins (**A**–**C**) and those only associated with platelets (**D**–**F**). (**A**,**D**) Top ten Biological processes enrichment assay of GO in terms of potential targets. (**B**,**E**) Cnetplot of GO analysis, and (**C**,**F**) Bubble chart showing the top 10 biological processes of GO terms.

**Figure 9:**
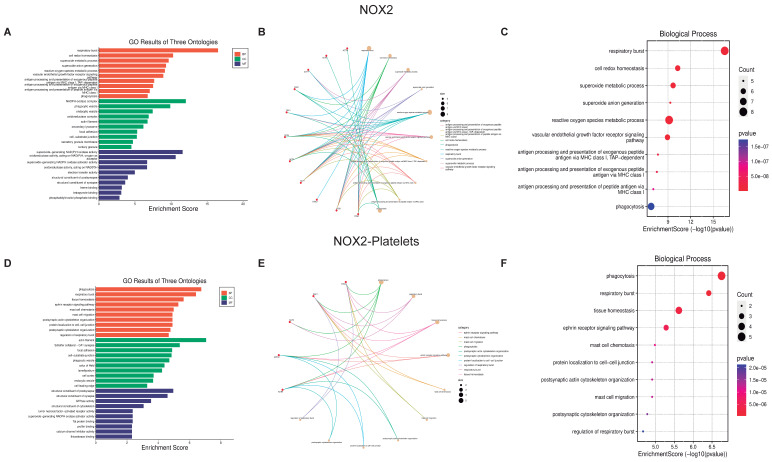
**Biological function assays of NOX2.** The graphs associated with NOX2 were constructed with all the proteins (**A**–**C**) and those only associated with platelets (**D**–**F**) are observed. (**A**,**D**) Top ten Biological processes enrichment assay of GO in terms of potential targets. (**B**,**E**) Cnetplot of GO analysis, and (**C**,**F**) Bubble chart showing the top 10 biological processes of GO terms.

The ten most critical biological processes were selected as they have the largest number of proteins and interactions these were analyzed using the previously mentioned graphics (GO Three Ontologies, Cnetplot, and Biological Processes Enrichment Score dot-plot) (Figure 8 and Figure 9). All analyses were performed for cellular components (Supplementary Figures S7 and S9) and molecular function (Supplementary Figures S8 and S10). Cnetplot shows that although there are fewer proteins compared to PDI, all of these are intimately related to all biological processes; therefore, the stimulation of a protein triggers a large number of biological processes. Activation of the NOX isoforms can regulate 14 proteins that in turn regulate more than 360 platelet proteins, from which a large number of biological processes that are vital for platelet function are modulated.

## 4. Discussion

We aimed to evaluate the interplay of NOX and PDI in platelet function by analyzing the main processes where the two enzymes participate during platelet activation and aggregation. The interaction network plots show a large number of protein interactions, where those proteins highly interacting with others were located in the middle of the plot while those with lower interactions were present in the borders. These results mean that if a protein is affected, depending on its interacting capacity, could have minor or major repercussions on many other proteins which can be associated with the development of dysfunctional processes involved in many diseases. PDI members’ interactions shown in Table 1 demonstrate the prevalence of PDI activity in several biological processes, e.g., protein folding [39], cellular viability [40], normal cellular physiology [41], disulfide interchange, chaperone [42], cytoskeleton organization [43], and redox regulation [44]. Then we confirmed with the Chord Plots that the PDIA3 isoform has a role in ER stress [45], protein folding processes [46], and cell–substrate adhesion [47]. Of relevance, integrins activation and PDI isoforms are connected in platelets, confirming that activation of integrins at the platelet membrane necessary for platelet adhesion and aggregation are connected to PDI activity [48]. Overall, the data support the importance of this enzyme for platelet physiology and function. In addition, our data in Figure 3 strongly support that activating the PDI isoforms P4HB, PDIA3, and PDIA6 is vital for the correct platelet activity in the organism. Hence, PDI is a relevant target for designing and studying antiplatelet drugs by analyzing different protein signaling pathways and their participation in disease onset and progression [49].

Several important processes for the correct function of platelets are related to the NOX function. NOX2 affects lamellipodium formation as well as cytoskeleton rearrangement and cell adhesion function [50]. Platelets, due to NOX, responding to environmental stimuli produce and release ROS, such as O_2_^.−^ and H_2_O_2_. Indeed, NOX led to an increase in oxidative stress affecting platelet mitochondrial function, and cell activation capacity [51]. The latter processes share their capacity to be regulated by PDI confirming the relevance of the interactions between PDI and NOX for the normal function and reactivity of platelets. The data presented in the current work correlate with those described by Laurindo et al. [52], in which he demonstrated the importance of the interaction between PDI and NOX in Vascular Smooth Muscle Cell (VSMC) migration into vessel neointima (Therapeutic target for atherosclerosis).

**Figure 10:**
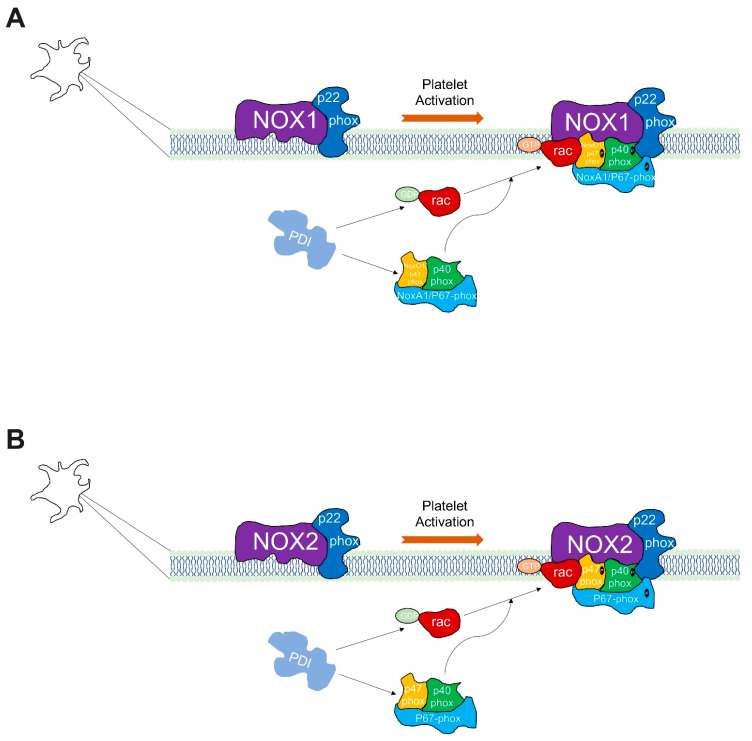
**Model of Protein disulfide isomerase-associated regulation of NADPH oxidase complexes in platelets.** PDI isoforms shown are intracellular proteins. Additionally, NOX1 and NOX2 active complexes are at the plasma membrane. NOX1 (**A**) and NOX2 (**B**) are associated with p22phox, and regulated by the small GTPase-Rac; this protein plays a pivotal role in both NOX1 and NOX2 activation orchestrating the assembly of the active enzyme complex. For its activation, the NOX1 enzyme complex requires the assembly of NOXO1/p47phox and NOXA1/p67phox (**A**). The NOX2 enzyme complex requires more proteins for binding, such as p22phox, p47phox, p67phox, and p40phox (**B**).

NOX active complex requires the formation of heterodimers with cytosolic and membrane proteins, some of them acting as active complex regulatory subunits such as p22phox. Both NOX1 and NOX2 are further dependent on association with a cytosolic ‘activator’ subunit, p67phox. The interaction of p67phox with protein domains on the intracellular loops of the NOX induces a conformational change necessary for the electron flow; in addition, the cytosolic subunit p47phox has an organizer role aiding in the correct migration to the membrane. Finally, a GTPase (Rac1 or Rac2) interacting with p67phox induces a conformational change in p67phox required for NOX activation. Several groups report an important role of PDI in the migration of p47phox and the cytosolic subunits’ correct interaction with gp91phox and p22phox at the membrane for O_2_^.−^ formation by NOX [32,33,53]. Importantly, the effects of PDI do not involve any transcriptional modifications of NOX subunits. The results shown in Figure 9 and Figure 10 for platelet ROS formation and the impact that either PDI or NOX isoforms have on platelet function, support the interactions of both proteins and are following the literature [54,55]. In general, the most relevant biological processes vital for the regulation of platelet function include the regulation of mitochondrial function, apoptosis, and oxidative stress. Mitochondria damage or dysfunction results in greatly attenuated platelet survival and increased risk for thrombovascular events. Also in platelet activation, the mitochondria regulate the increase in intracellular calcium, increase in the production of ROS, and exposure to phosphatidylserine [56]. Regarding apoptosis, recent studies have identified apoptosis in the anucleate platelet, where blebbing, exposure of phosphatidylserine, and release of microparticles or exosomes stands out [57], the platelet contains various anti- or pro-apoptotic proteins, such as BCL-2, BAK, and BAX. When platelet activation occurs causes the activation of BAK and BAX, initiating the subsequent release of mitochondrial components such as cytochrome c through pores in the mitochondrial membrane [58]. On the other hand, oxidative stress and persistent stimuli lead to excessive platelet activation, resulting in a platelet procoagulant phenotype and apoptosis, both accounting for the high thrombotic risk in oxidative stress-related diseases [5].

## 5. Conclusions

Our study reveals the direct correlation of PDI and NOX in platelets starting from the complexity of cell proteins through to the analysis of how each protein impacts platelet function. The data follow several experimental reports from our labs [34,59,60,61]. We conclude that the five enzymes analyzed in platelets regulate more than 200 proteins, which in turn can interact with more than the other 2600 proteins involved in relevant biological processes. Overall, we conclude that the study of Platelet Redox Signaling is vital to establishing platelet function and the subsequent design and generation of inhibitors and therapeutic strategies that can contribute to the modulation of thrombotic and cardiovascular diseases.

## Figures and Tables

**Table 1:** Protein Interaction.

Protein	Interaction	Node	Edges
P4HB	All	8185	20,975
	Platelet	1713	3842
PDIA3	All	7988	18,831
	Platelet	1859	4001
PDIA6	All	7542	17,855
	Platelet	1868	4299
NOX1	All	480	499
	Platelet	158	161
NOX2	All	1181	1349
	Platelet	242	254

**Table 2:** Biological Processes.

Protein	Interaction	Count
P4HB	All	284
	Platelet	181
PDIA3	All	309
	Platelet	207
PDIA6	All	177
	Platelet	105
NOX1	All	14
	Platelet	4
NOX2	All	40
	Platelet	27

## Data Availability

The data presented in this study are available in the main text, figures, tables and Supplementary Material.

## Supplementary Materials

The following supporting information can be downloaded at: https://www.mdpi.com/article/10.3390/biom13050848/s1, Figure S1: Cellular component assays of PH4HB isomerase; Figure S2: Molecular function assays of PH4HB isomerase; Figure S3: Cellular component assays of PDIA3 isomerase; Figure S4: Molecular function assays of PDIA3 isomerase; Figure S5: Cellular component assays of PDIA6 isomerase; Figure S6: Molecular function assays of PDIA6 isomerase; Figure S7: Cellular component assays of NOX1; Figure S8: Molecular function assays of NOX1; Figure S9: Cellular component assays of NOX2; Figure S10. Molecular function assays of NOX2.

## Abbreviations

^.^NO	Nitric Oxide
-CXXC-	catalytic vicinal active site thiols
AAR2	AAR2 Splicing Factor
BP	Biological Processes
CC	Cellular Component
CUL1	Cullin 1
CUL2	Cullin 2
CUL3	Cullin 3
DUOX1	Dual Oxidase 1
DUOX2	Dual Oxidase 2
ER	Endoplasmic Reticulum
ERP29	Endoplasmic Reticulum Protein 29
ERP44	Endoplasmic Reticulum Protein 44
GO	Gene Ontology
H_2_O_2_	Hydrogen Peroxide
KEGG	Kyoto Encyclopedia of Genes and Genomes
KRAS	KRAS Proto-Oncogene, GTPase
MF	Molecular Functions
NOX	NADPH oxidase
NOX1	NADPH Oxidase 1
NOX2	NADPH Oxidase 2
NOX3	NADPH Oxidase 3
NOX4	NADPH Oxidase 4
NOX5	NADPH Oxidase 5
O_2_^.−^	Superoxide
P4HB	Prolyl 4-Hydroxylase Subunit Beta
PDI	Protein Disulphide Isomerase
PDIA3	Protein Disulfide Isomerase Family A Member 3
PDIA4	Protein Disulfide Isomerase Family A Member 4
PDIA6	Protein Disulfide Isomerase Family A Member 6
RAC1	Rac Family Small GTPase 1
RAC2	Rac Family Small GTPase 2
ROS	Reactive Oxygen Species
SOD1	Superoxide Dismutase 1
TMX3	Thioredoxin Related Transmembrane Protein 3
TNFRSF1A	Tumor Necrosis Factor Receptor 1
UBE2M	Ubiquitin Conjugating Enzyme E2 M
