# Supplementary material for: An Overview of Two Old Friends Associated with Platelet Redox Signaling, the Protein Disulfide Isomerase and NADPH Oxidase
Source: Biomolecules. 2023 May 17;13(5):848. doi: 10.3390/biom13050848 (PMC10216113; doi:10.3390/biom13050848)
Supplement: Supplementary file 1 [file biomolecules-13-00848-s001.zip › biomolecules-2122105-supplementary.pdf]

**Supplementary Figure S1. Cellular component assays of PH4HB isomerase.** The graphs associated with PH4HB isomerase constructed with all the proteins (A to C) and those only associated with platelets (D to F) are shown. (A and D) Top ten cellular component enrichment assay of GO in terms of potential targets. (B and E) Cnetplot of GO analysis, and (C and F) Bubble chart showing the top 10 cellular components of GO terms.

**Supplementary Figure S2. Molecular function assays of PH4HB isomerase.** The graphs associated with PH4HB isomerase constructed with all the proteins (A to C) and those only associated with platelets (D to F) are plotted. (A and D) Top ten molecular function enrichment assay of GO in terms of potential targets. (B and E) Cnetplot of GO analysis, and (C and F) Bubble chart showing the top 10 molecular functions of GO terms.

**Supplementary Figure S3. Cellular component assays of PDIA3 isomerase.** Graphs associated with PDAI3 isomerase constructed with all the proteins (A to C) and those only associated with platelets (D to F). (A and D) Top ten cellular component enrichment assay of GO in terms of potential targets. (B and E) Cnetplot of GO analysis, and (C and F) Bubble chart showing the top 10 cellular components of GO terms.

**Supplementary Figure S4. Molecular function assays of PDIA3 isomerase.** The graphs associated with PDIA3 isomerase constructed with all the proteins (A to C) and those only associated with platelets (D to F) are shown. (A and D) Top ten molecular function enrichment assay of GO in terms of potential targets. (B and E) Cnetplot of GO analysis, and (C and F) Bubble chart showing the top 10 molecular functions of GO terms.

**Supplementary Figure S5. Cellular component assays of PDIA6 isomerase.** Graphs associated with PDAI6 isomerase constructed with all the proteins (A to C) and those only associated with platelets (D to F) are observed. (A and D) Top ten cellular component enrichment assay of GO in terms of potential targets. (B and E) Cnetplot of GO analysis, and (C and F) Bubble chart showing the top 10 cellular components of GO terms.

**Supplementary Figure S6. Molecular function assays of PDIA6 isomerase.** The graphs associated with PDIA6 isomerase constructed with all the proteins (A to C) and those only associated with platelets (D to F) are shown. (A and D) Top ten molecular function enrichment assay of GO in terms of potential targets. (B and E) Cnetplot of GO analysis, and (C and F) Bubble chart showing the top 10 molecular functions of GO terms.

**Supplementary Figure S7. Cellular component assays of NOX1.** Graphs associated with NOX1 constructed with all the proteins (A to C) and those only associated with platelets (D to F) are plotted. (A and D) Top ten cellular component enrichment assay of GO in terms of potential targets. (B and E) Cnetplot of GO analysis, and (C and F) Bubble chart showing the top 10 cellular components of GO terms.

**Supplementary Figure S8. Molecular function assays of NOX1.** The graphs associated with NOX1 constructed with all the proteins (A to C) and those only associated with platelets (D to F) are represented. (A and D) Top ten molecular function enrichment assay of GO in terms of potential targets. (B and E) Cnetplot of GO analysis, and (C and F) Bubble chart showing the top 10 molecular functions of GO terms.

**Supplementary Figure S9. Cellular component assays of NOX2.** The graphs associated with NOX2 constructed with all the proteins (A to C) and those only associated with platelets (D to F) are shown. (A and D) Top ten cellular component enrichment assay of GO in terms of potential targets. (B and E) Cnetplot of GO analysis, and (C and F) Bubble chart showing the top 10 cellular components of GO terms.

**Supplementary Figure S10. Molecular function assays of NOX2.** The graphs associated with NOX2 constructed with all the proteins (A to C) and those only associated with platelets (D to F) are plotted. (A and D) Top ten molecular function enrichment assay of GO in terms of potential targets. (B and E) Cnetplot of GO analysis, and (C and F) Bubble chart showing the top 10 molecular functions of GO terms.
