# Supplementary figures and images for: An Overview of Two Old Friends Associated with Platelet Redox Signaling, the Protein Disulfide Isomerase and NADPH Oxidase
Source: Biomolecules. 2023 May 17;13(5):848. doi: 10.3390/biom13050848 (PMC10216113; doi:10.3390/biom13050848)

A

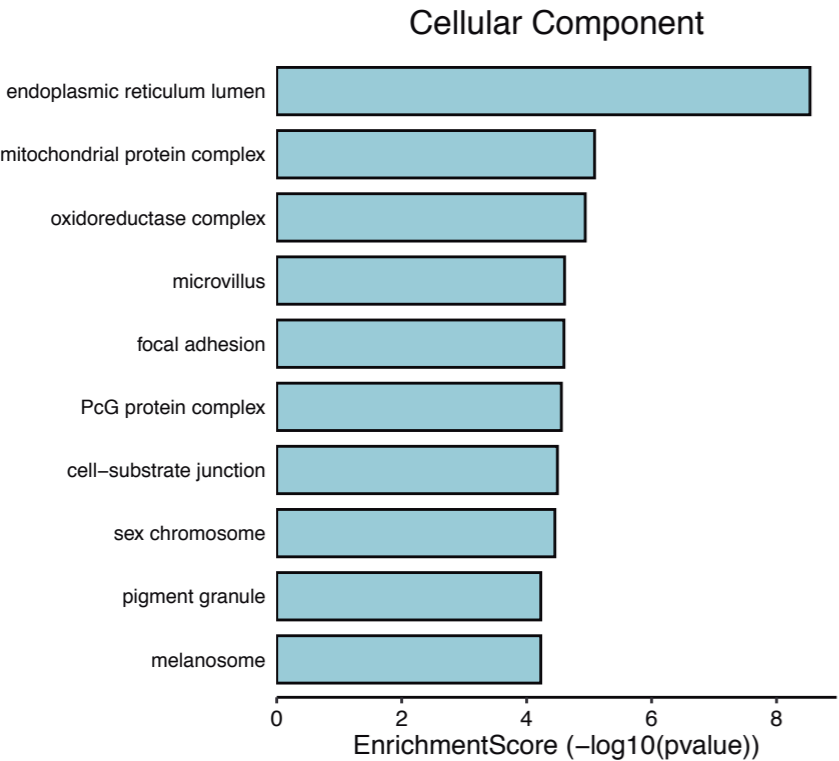

B

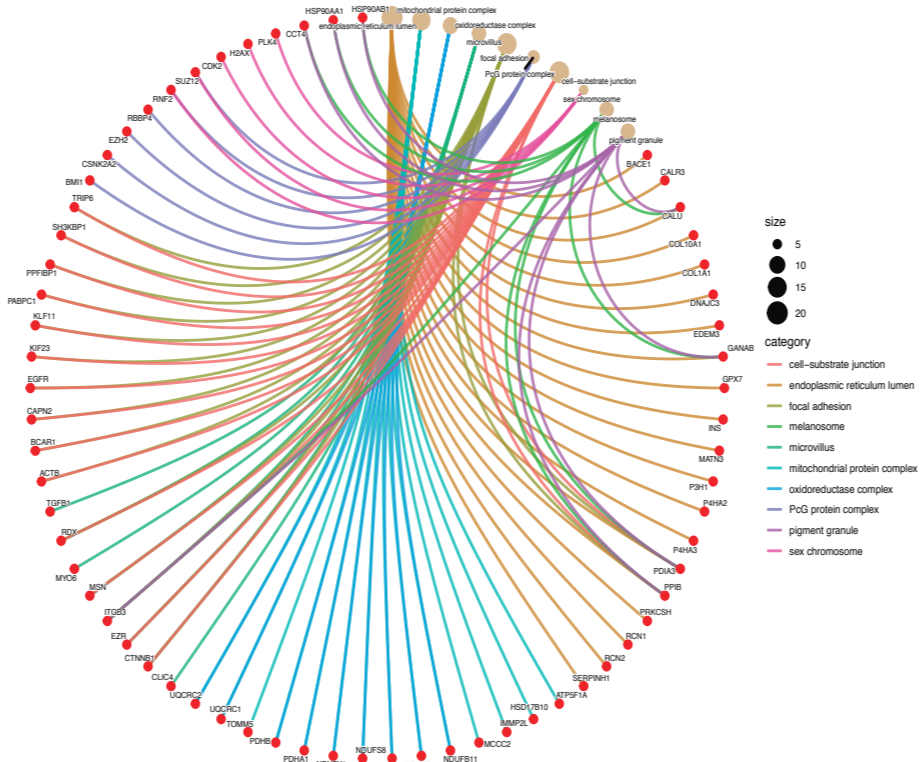

C

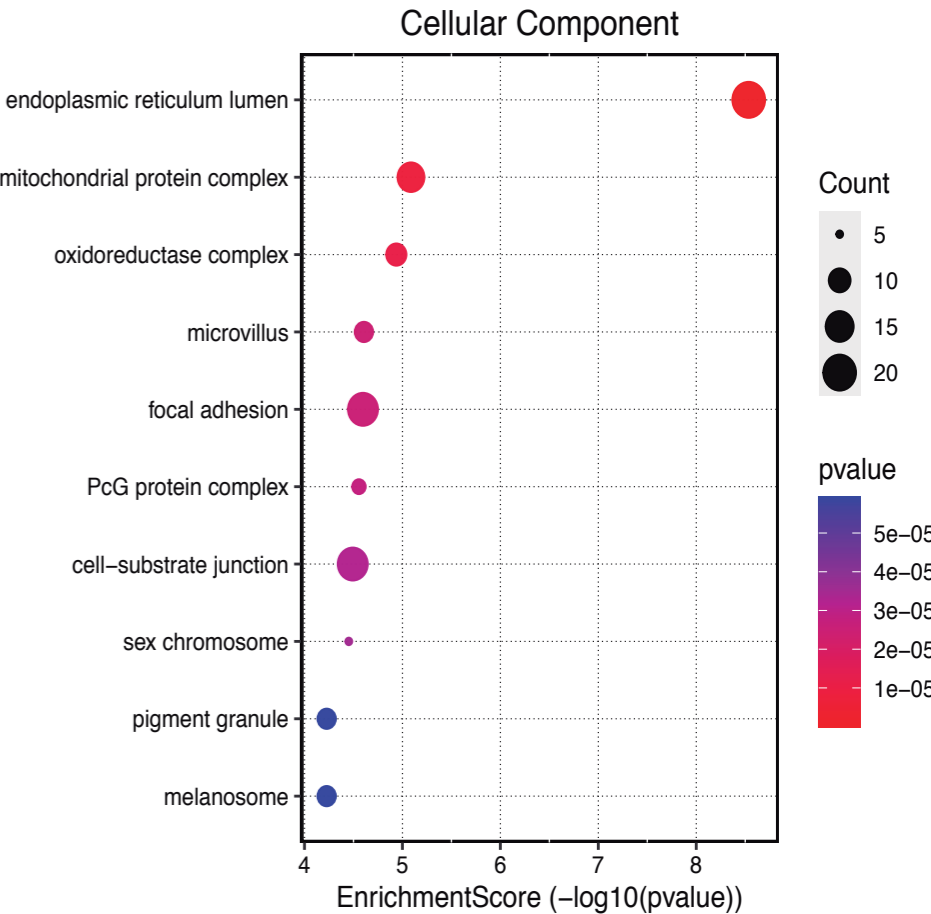

D

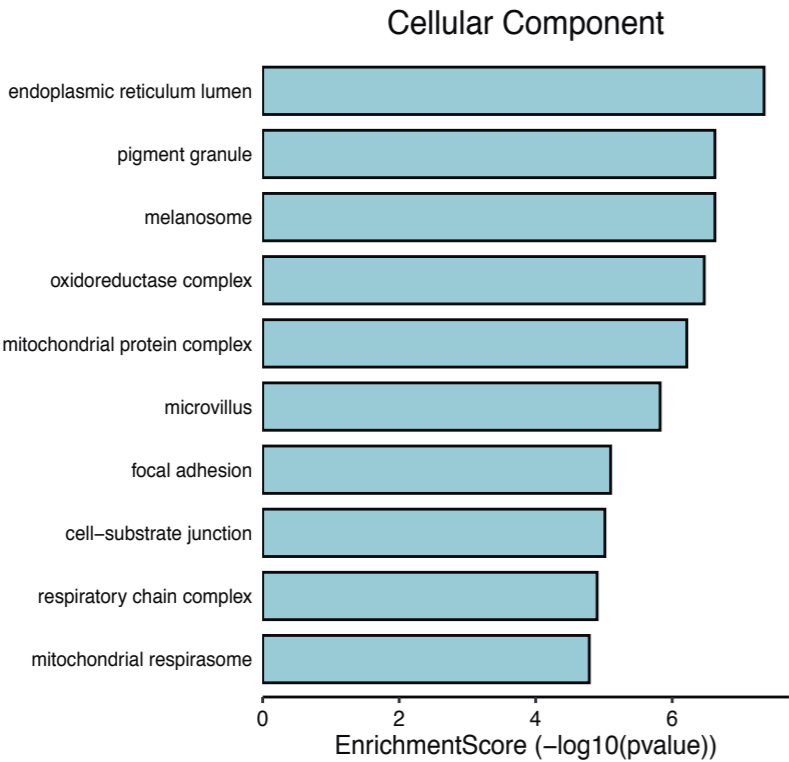

E

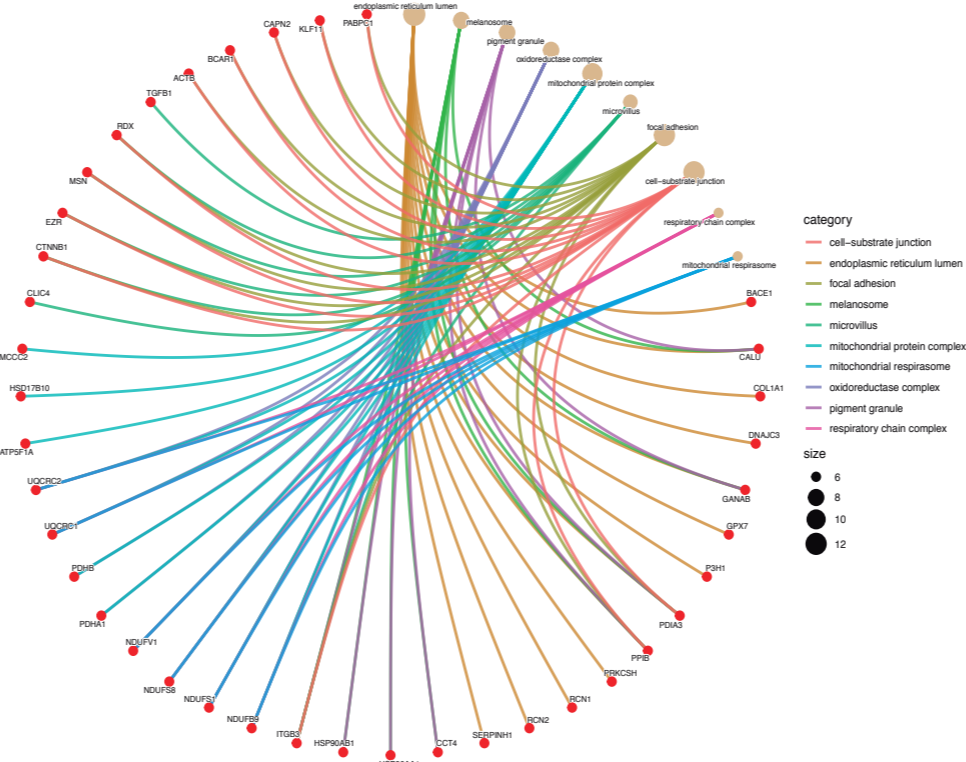

F

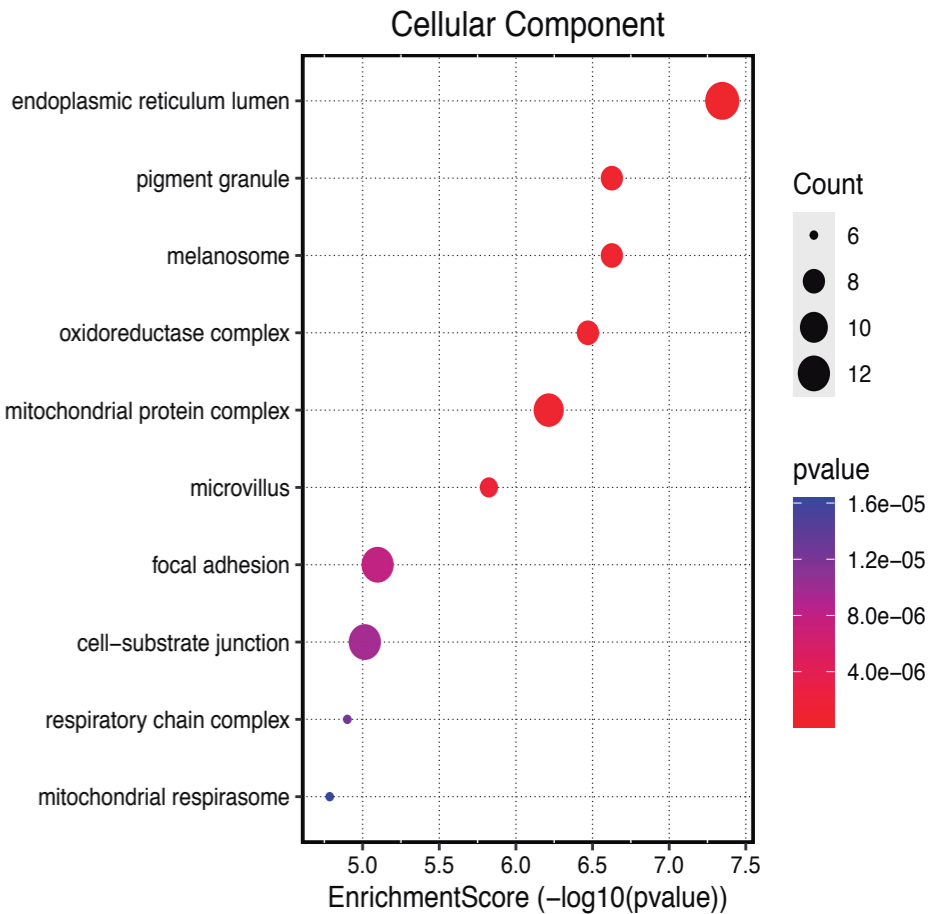

Supplement: Supplementary file 1 [file biomolecules-13-00848-s001.zip › Supplementary Figure S1.pdf]

A

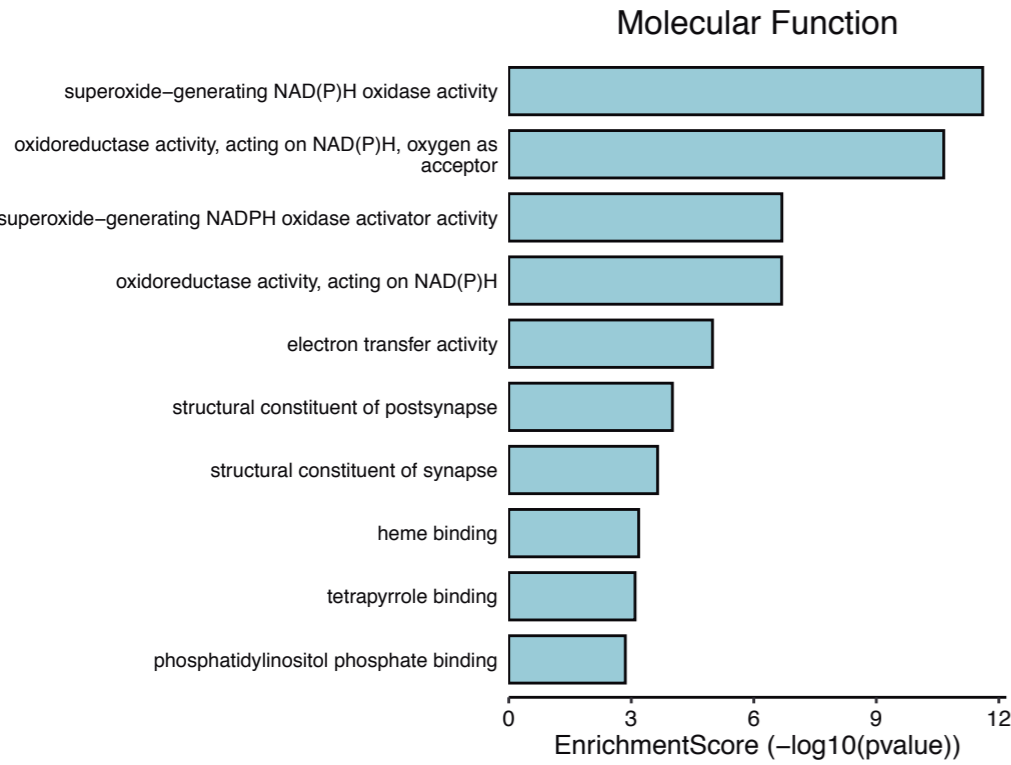

B

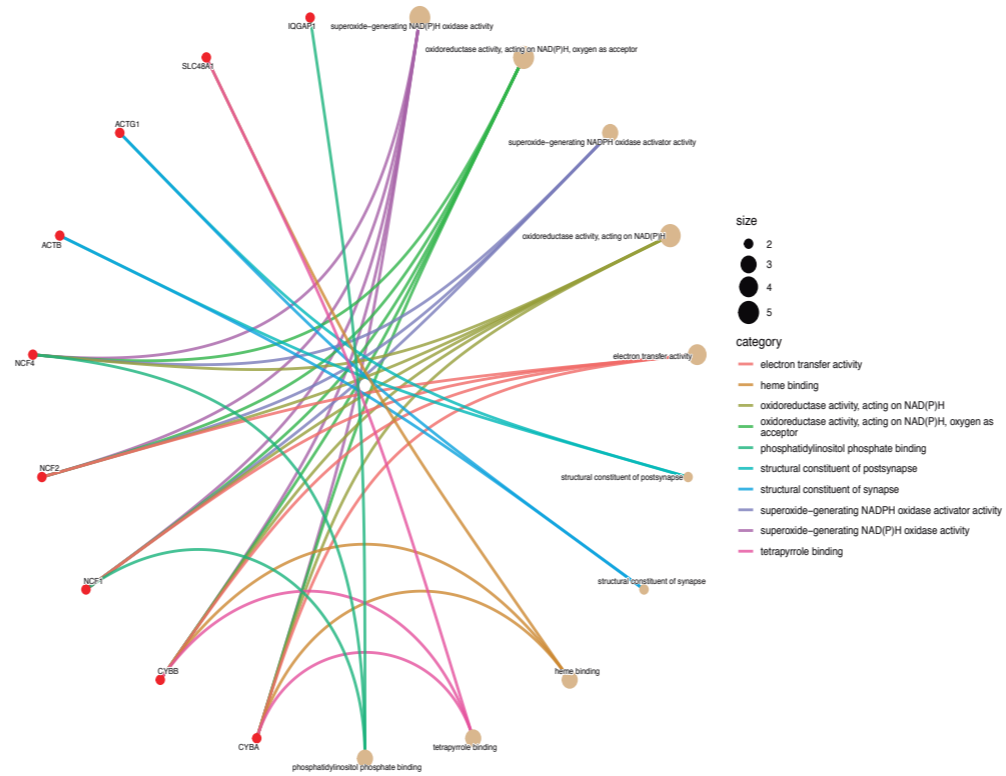

C

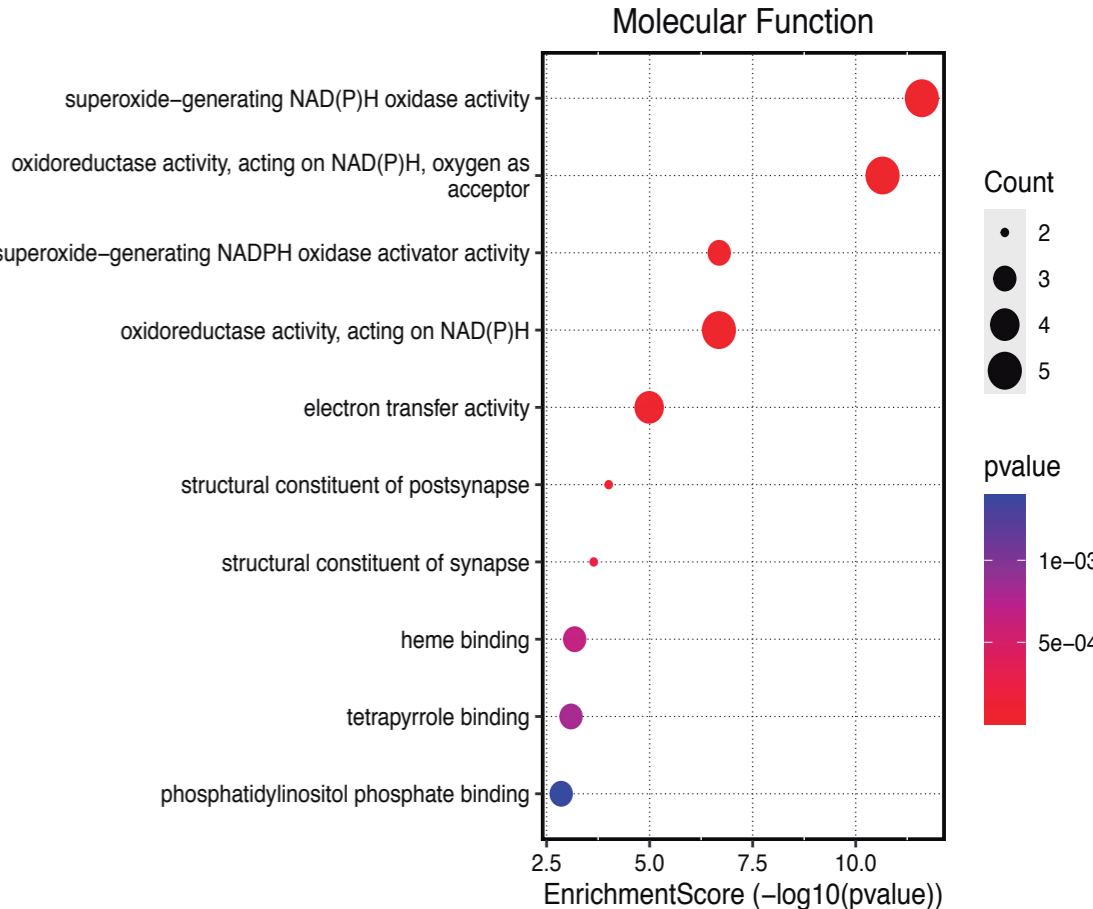

D

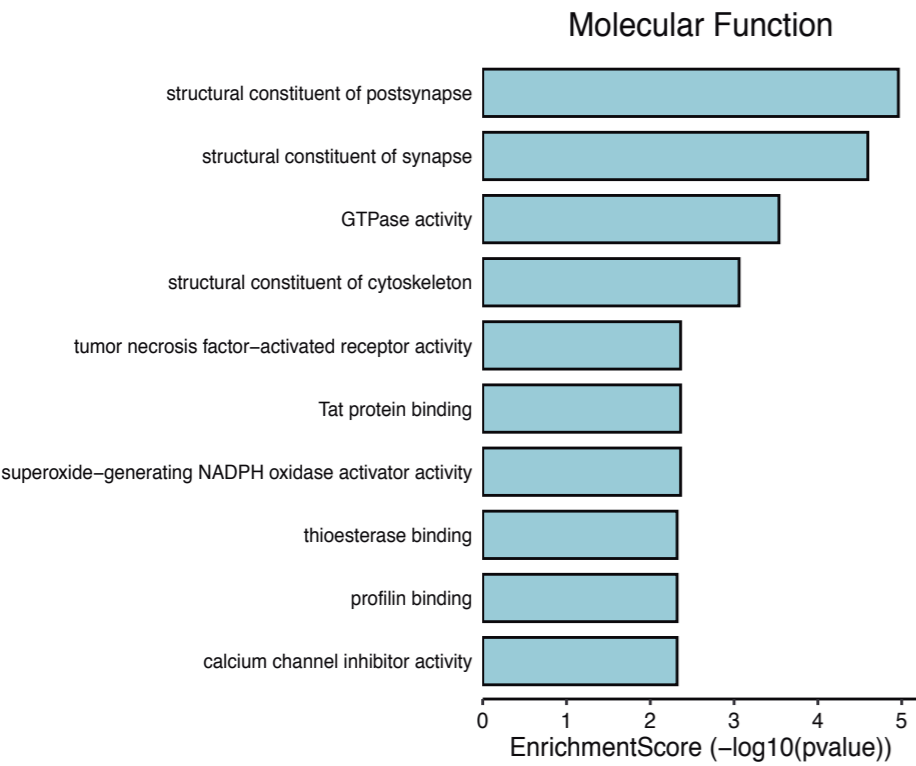

E

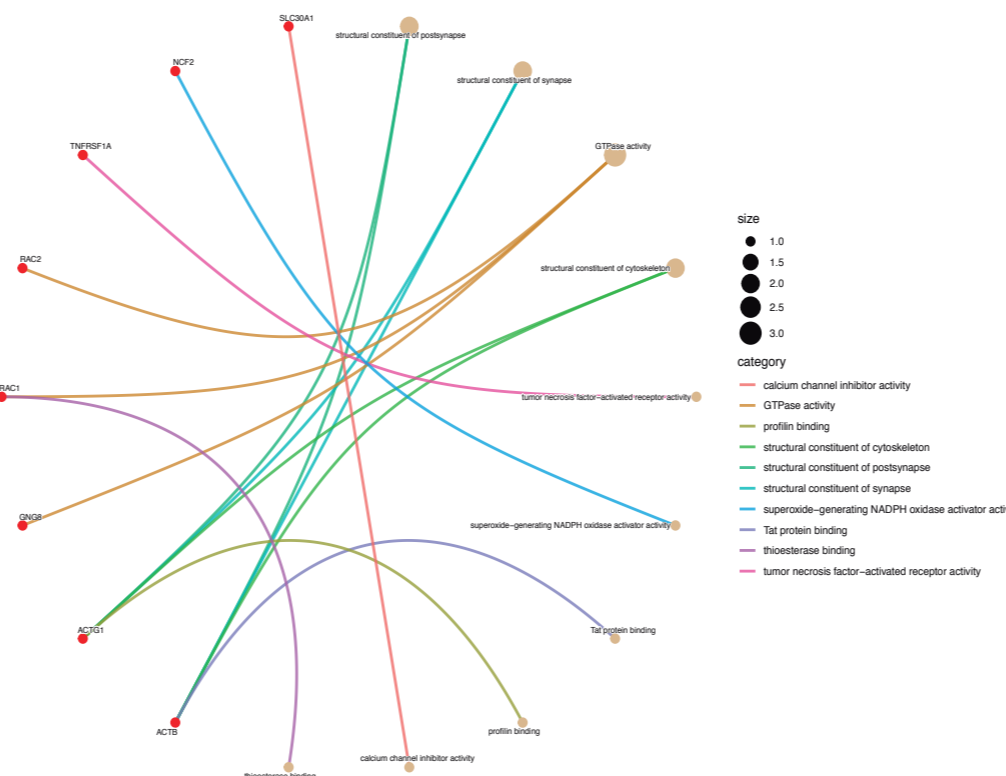

F

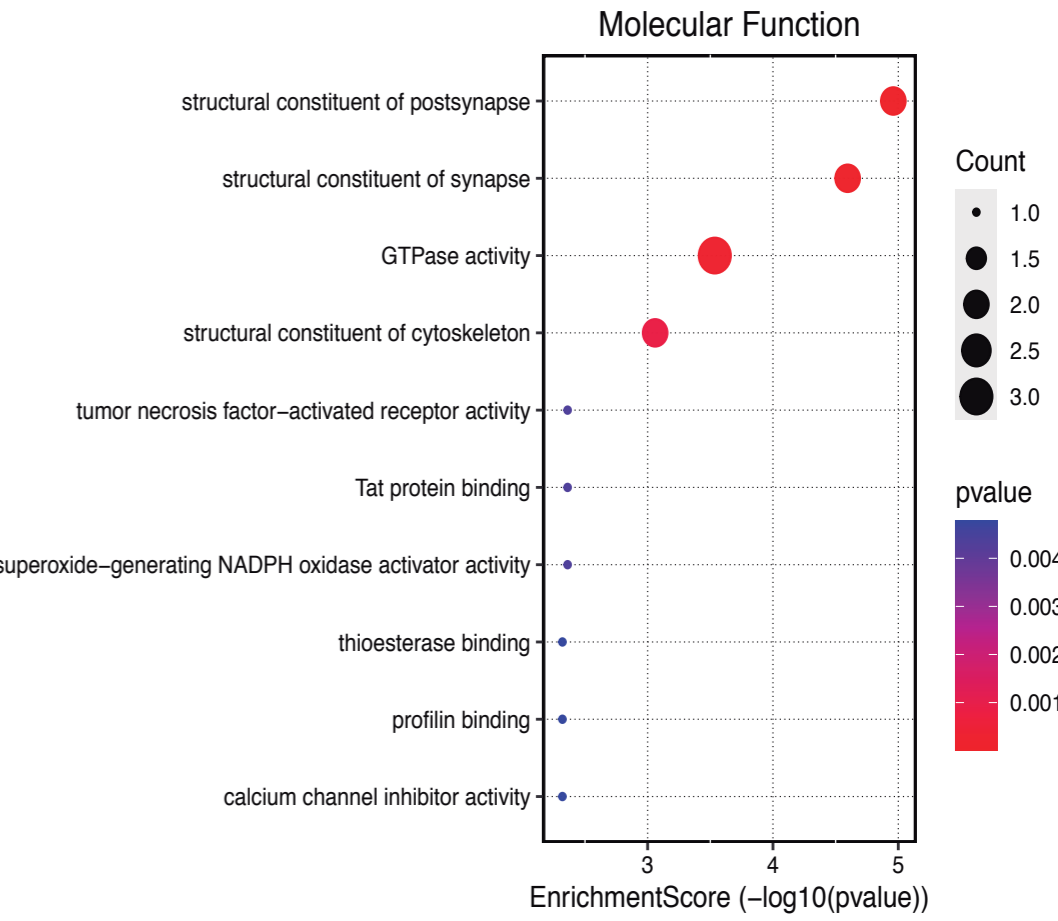

Supplement: Supplementary file 1 [file biomolecules-13-00848-s001.zip › Supplementary Figure S10.pdf]

A

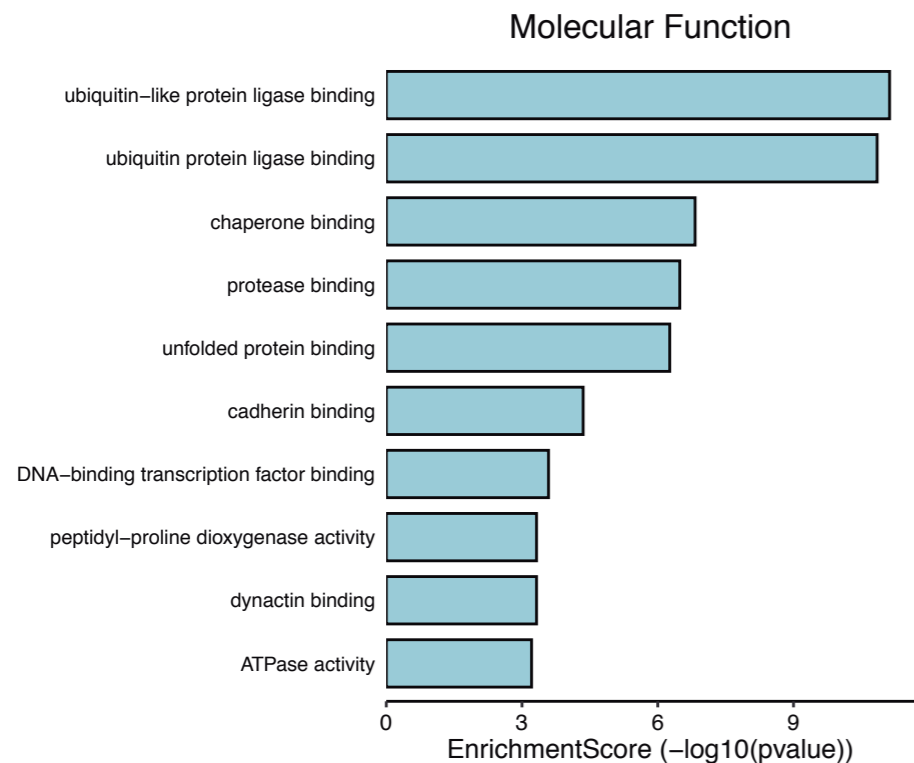

B

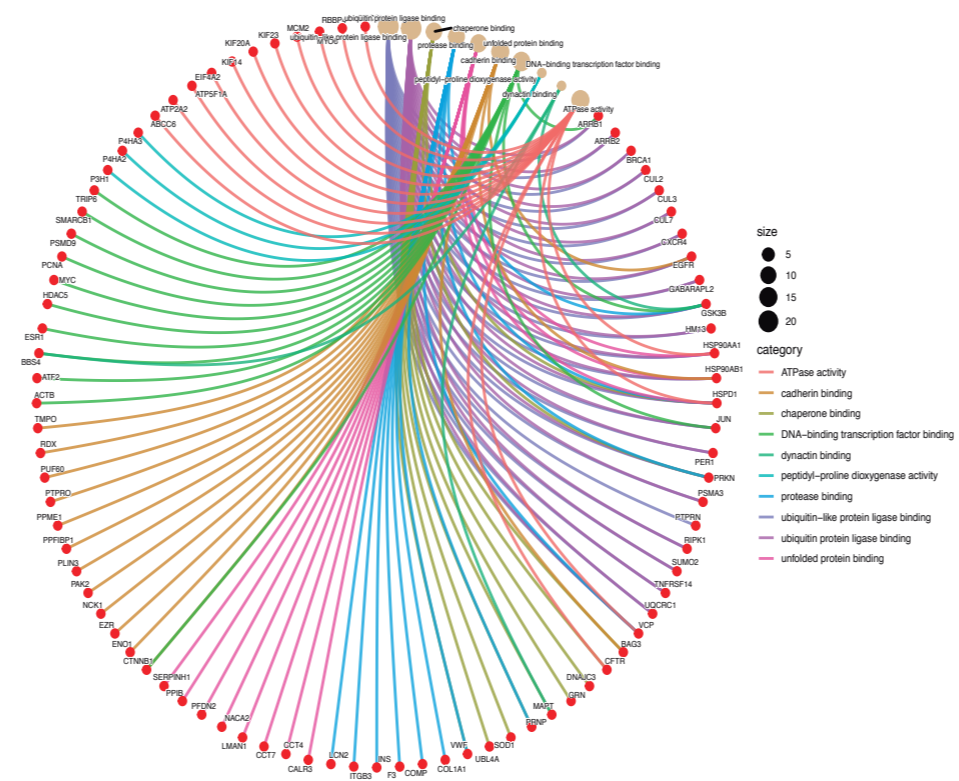

C

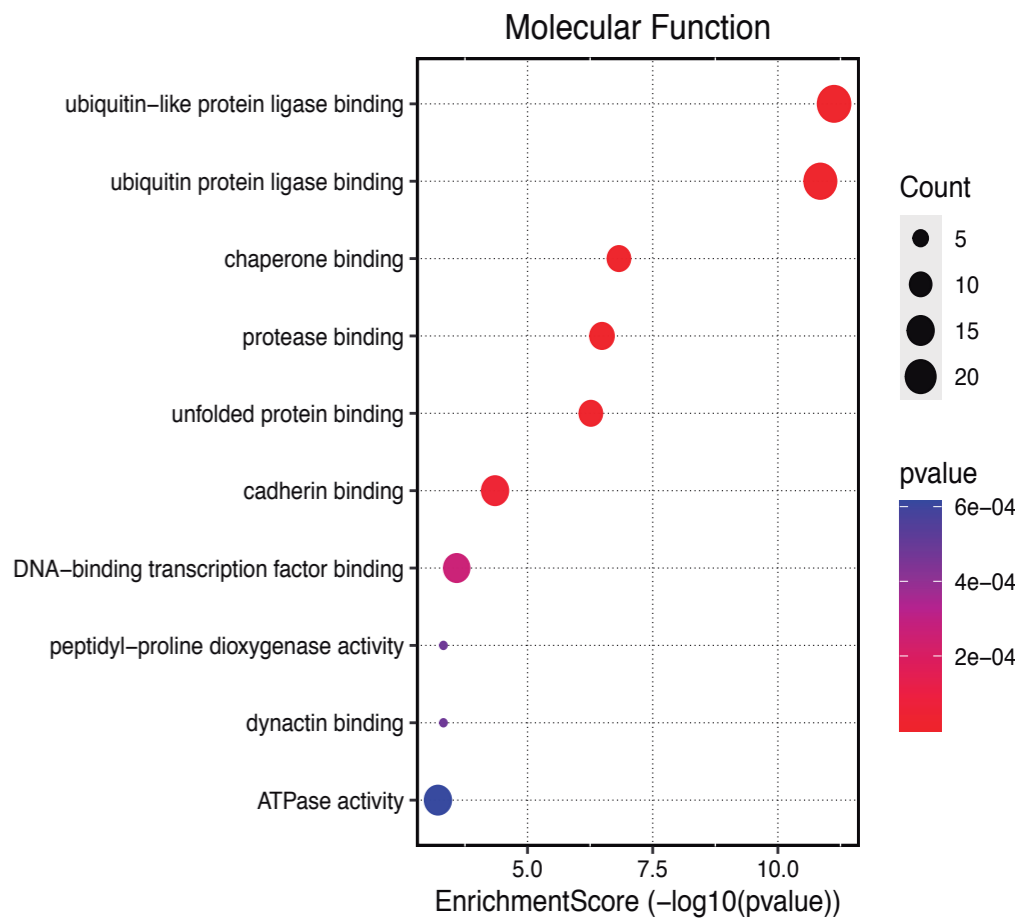

D

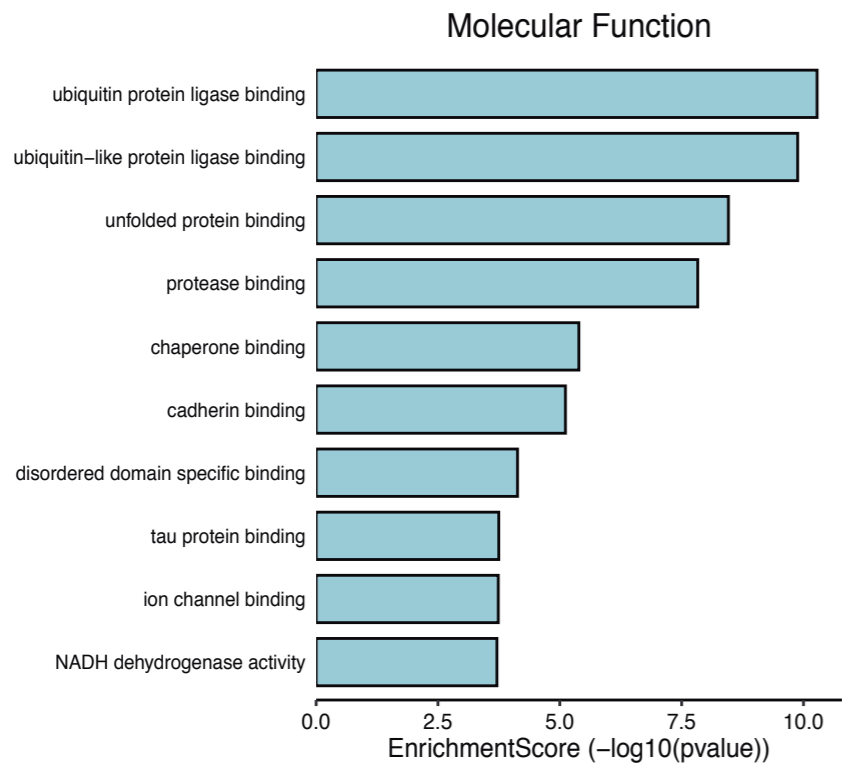

E

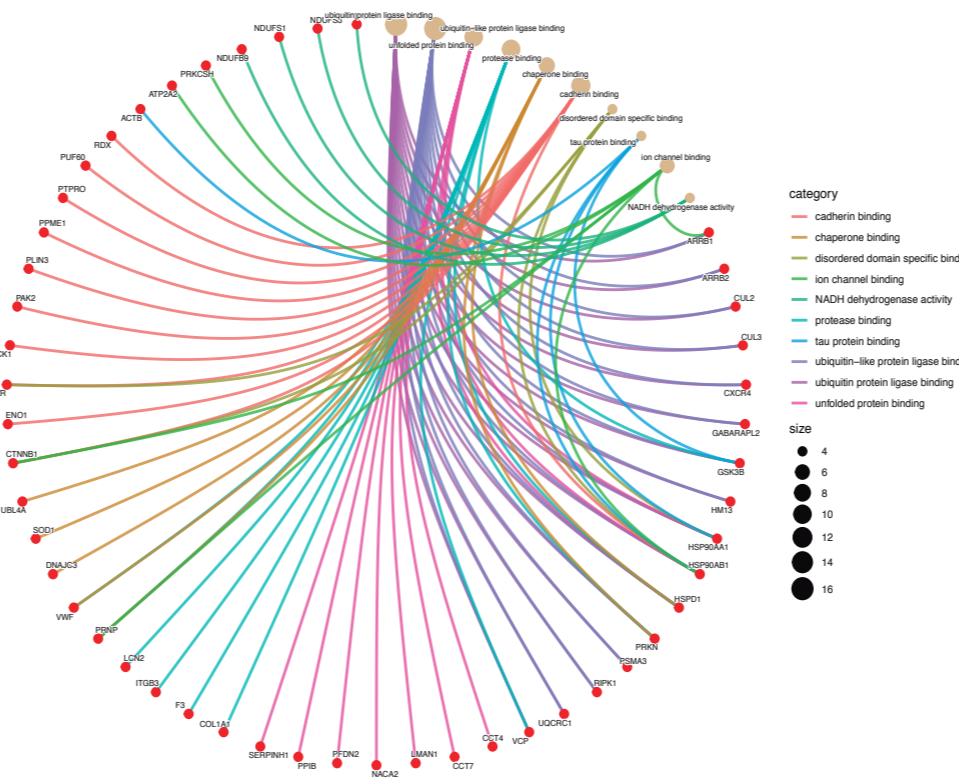

F

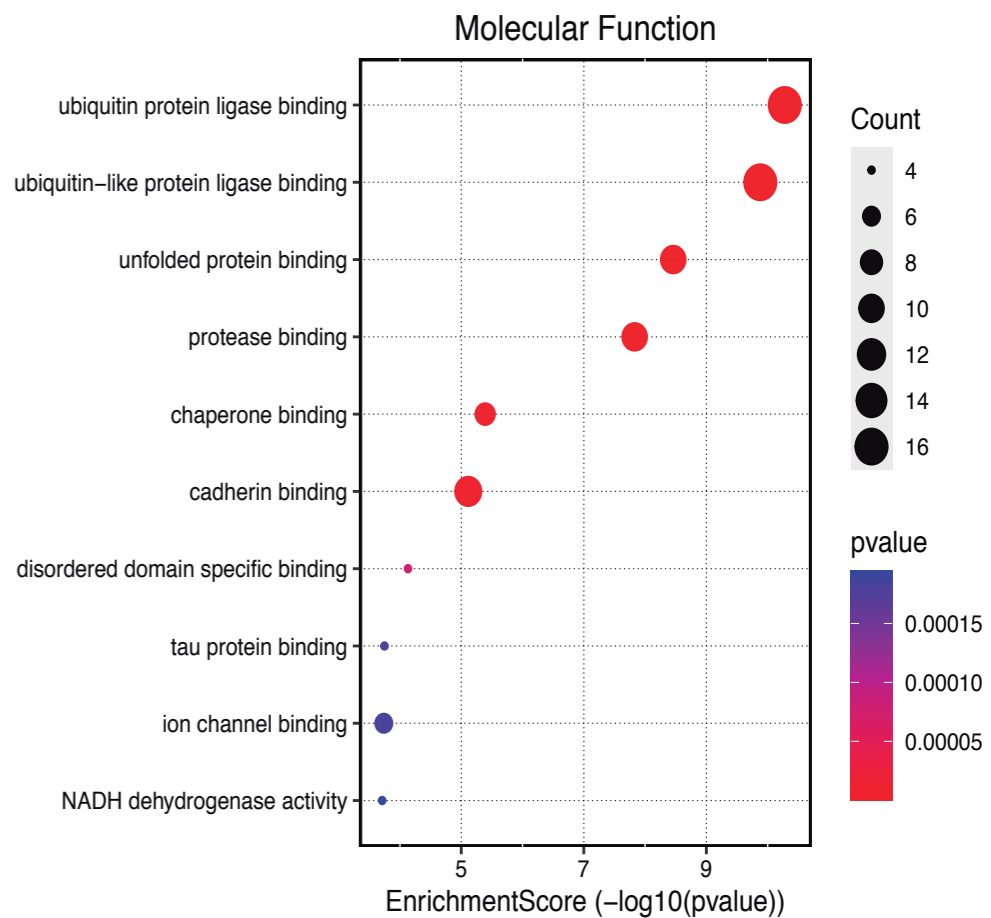

Supplement: Supplementary file 1 [file biomolecules-13-00848-s001.zip › Supplementary Figure S2.pdf]

PDIA3

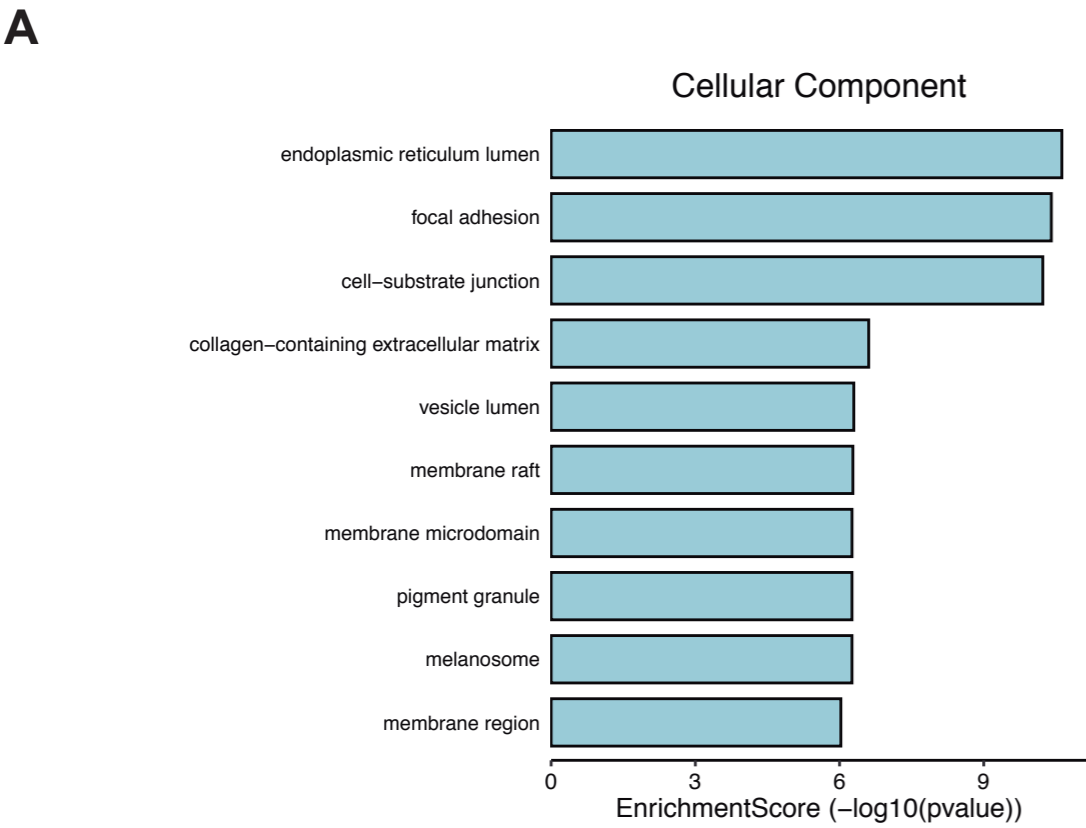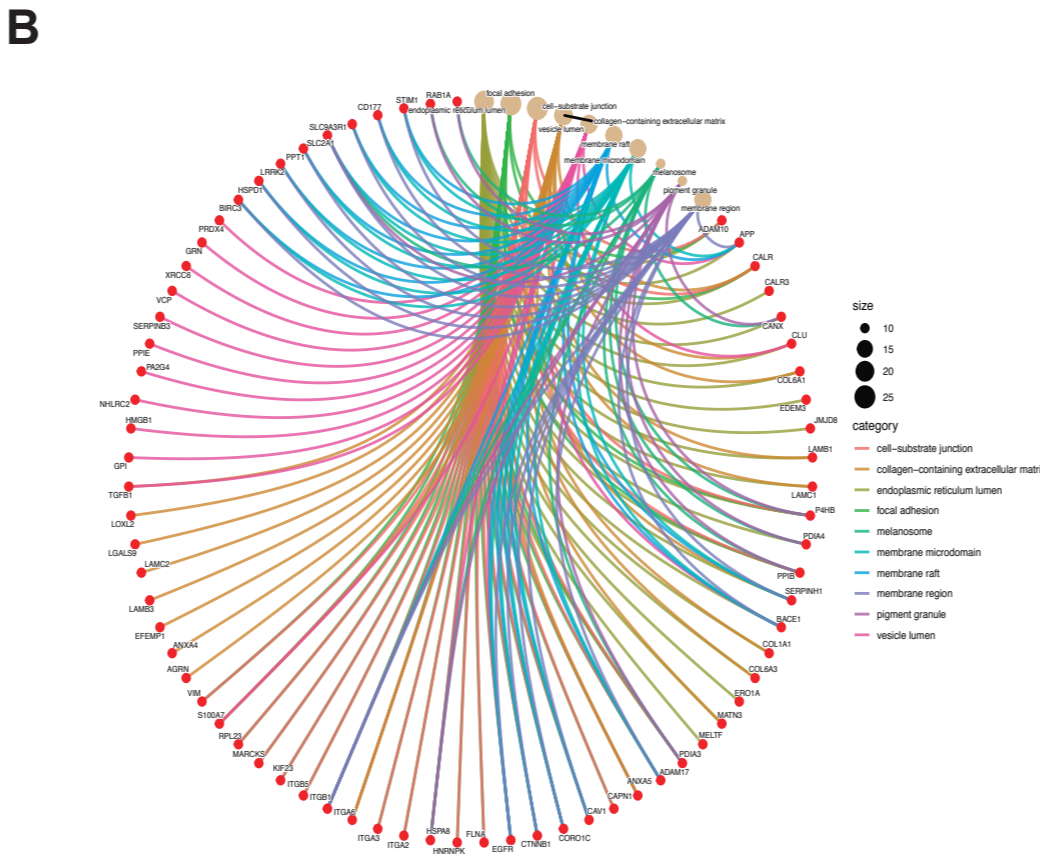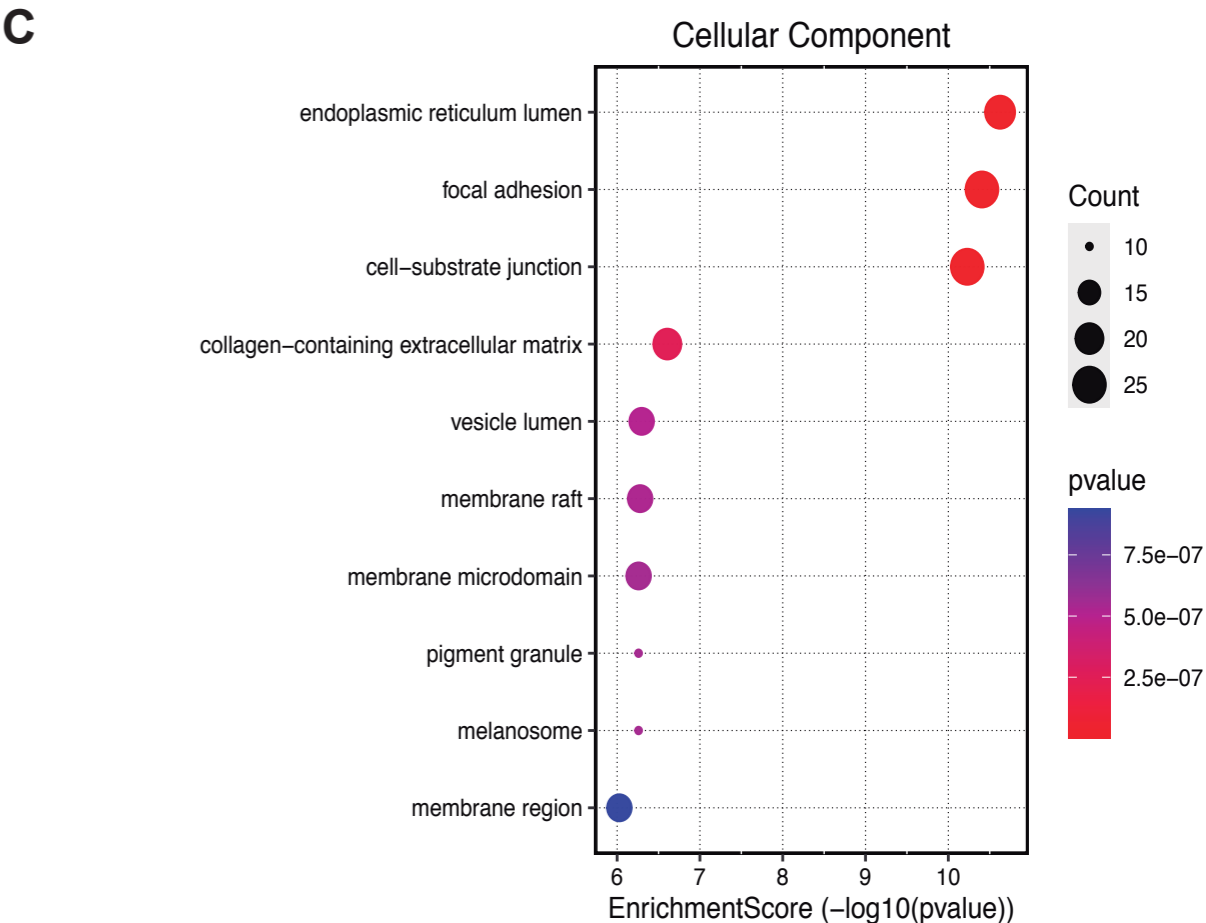

PDIA3-Platelets

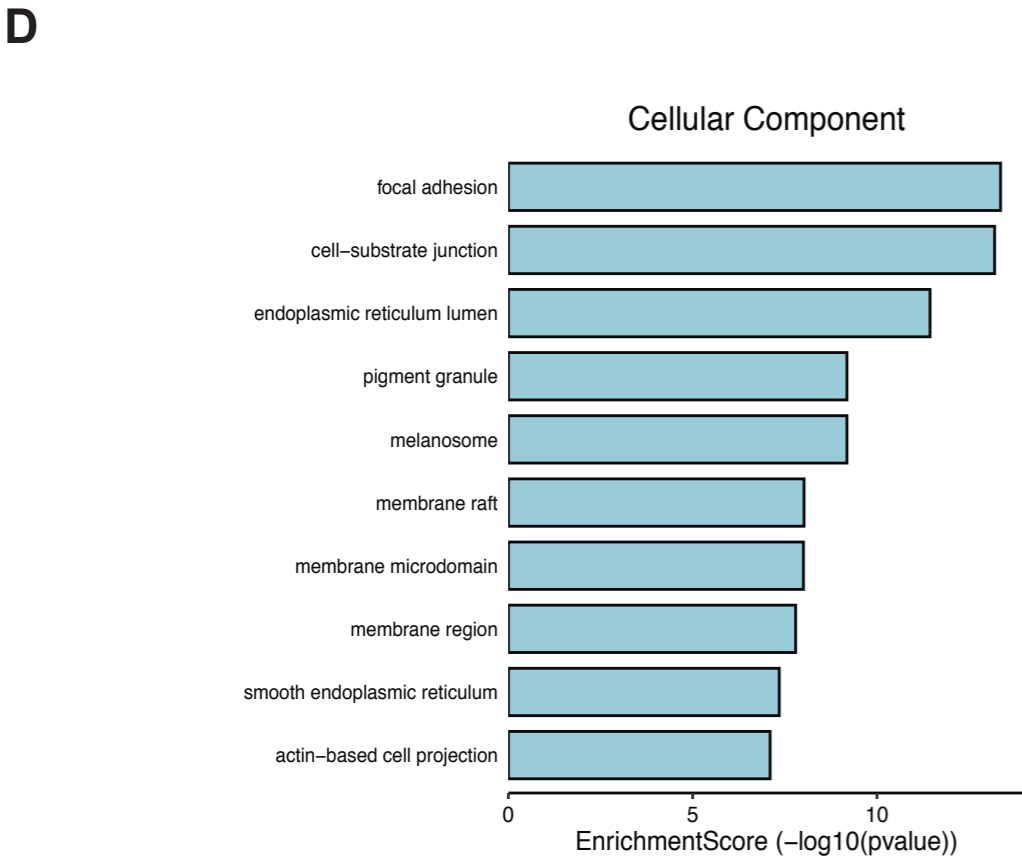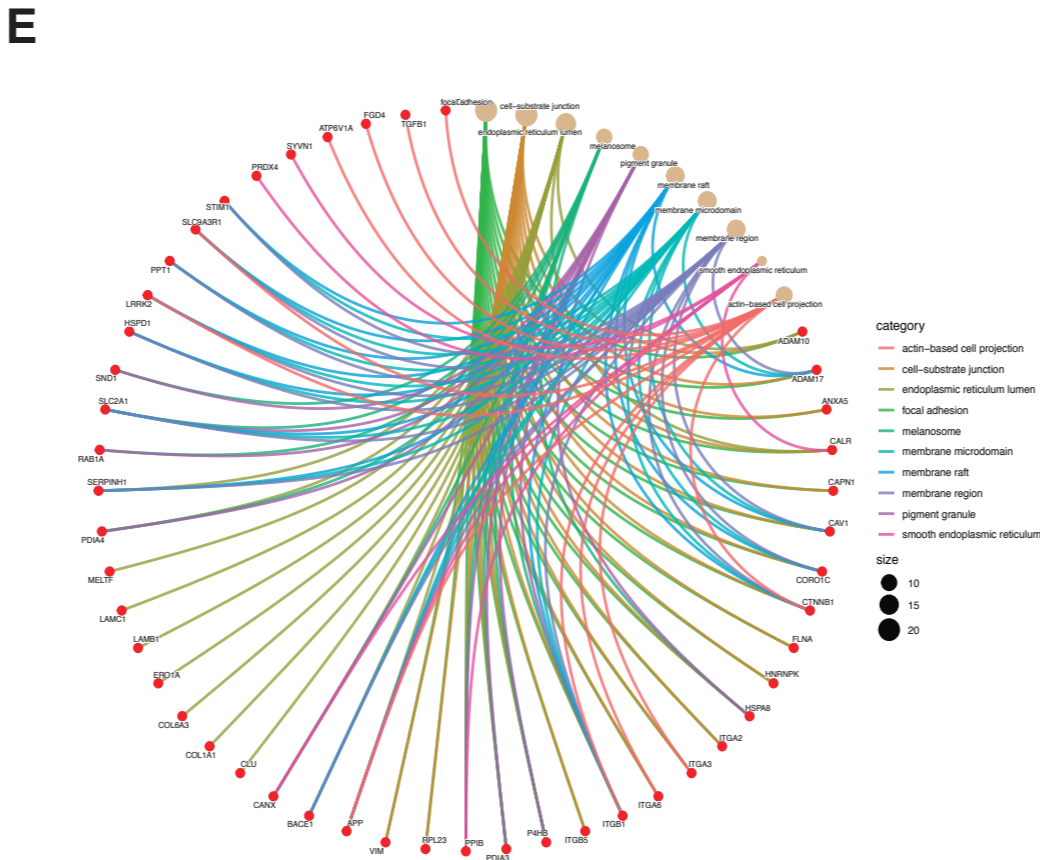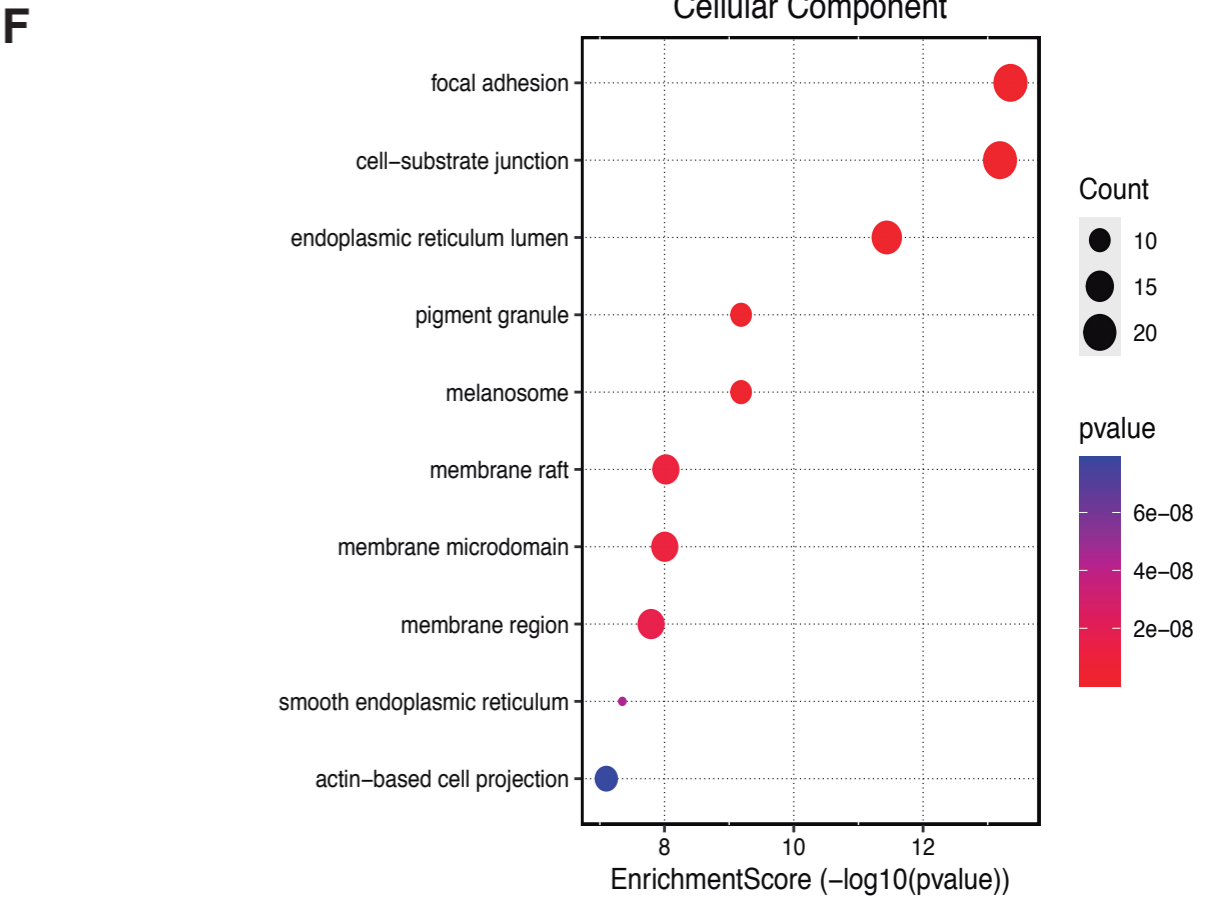

Supplement: Supplementary file 1 [file biomolecules-13-00848-s001.zip › Supplementary Figure S3.pdf]

PDIA3

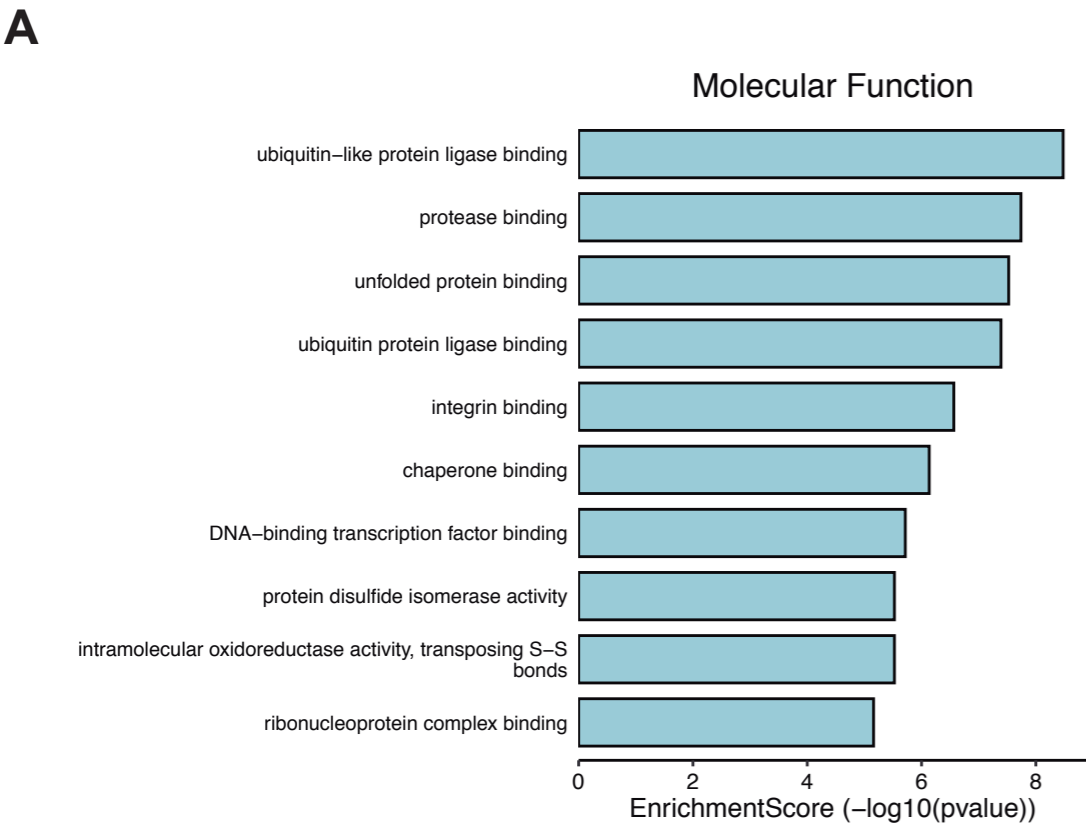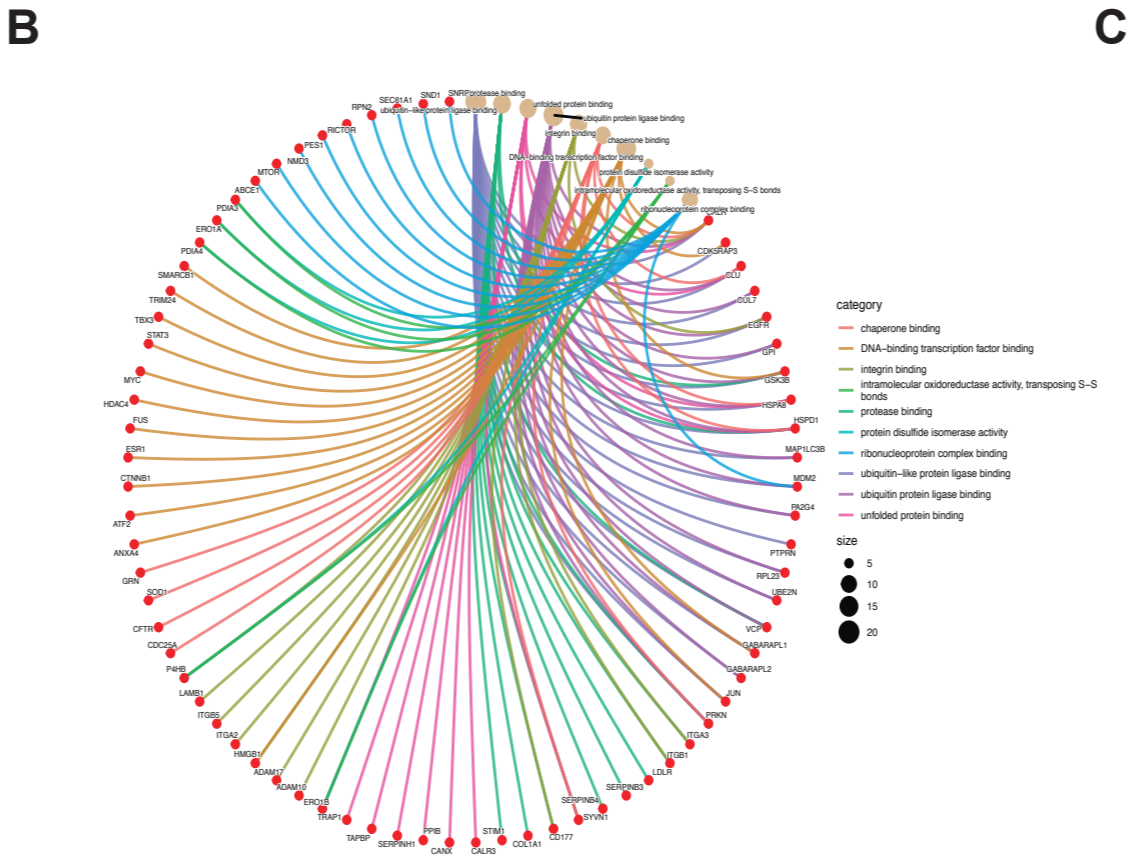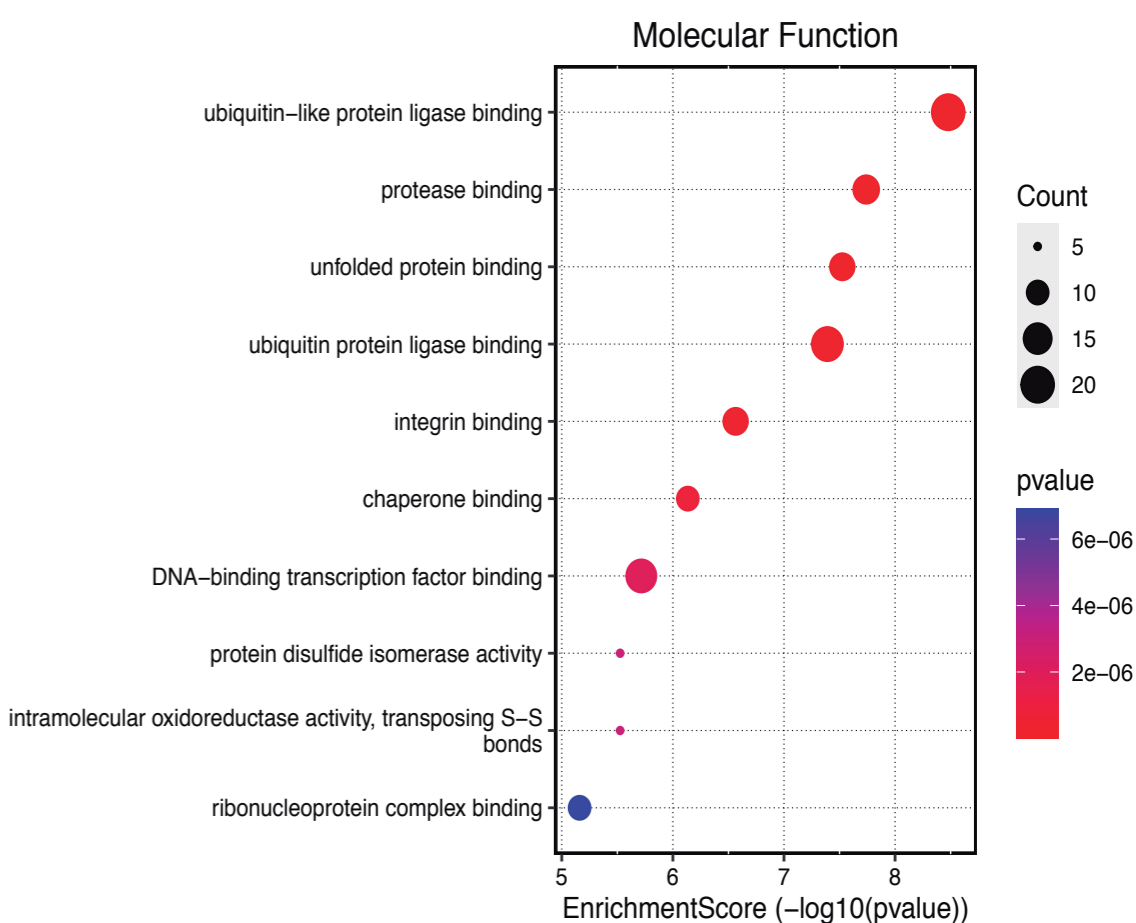

PDIA3-Platelets

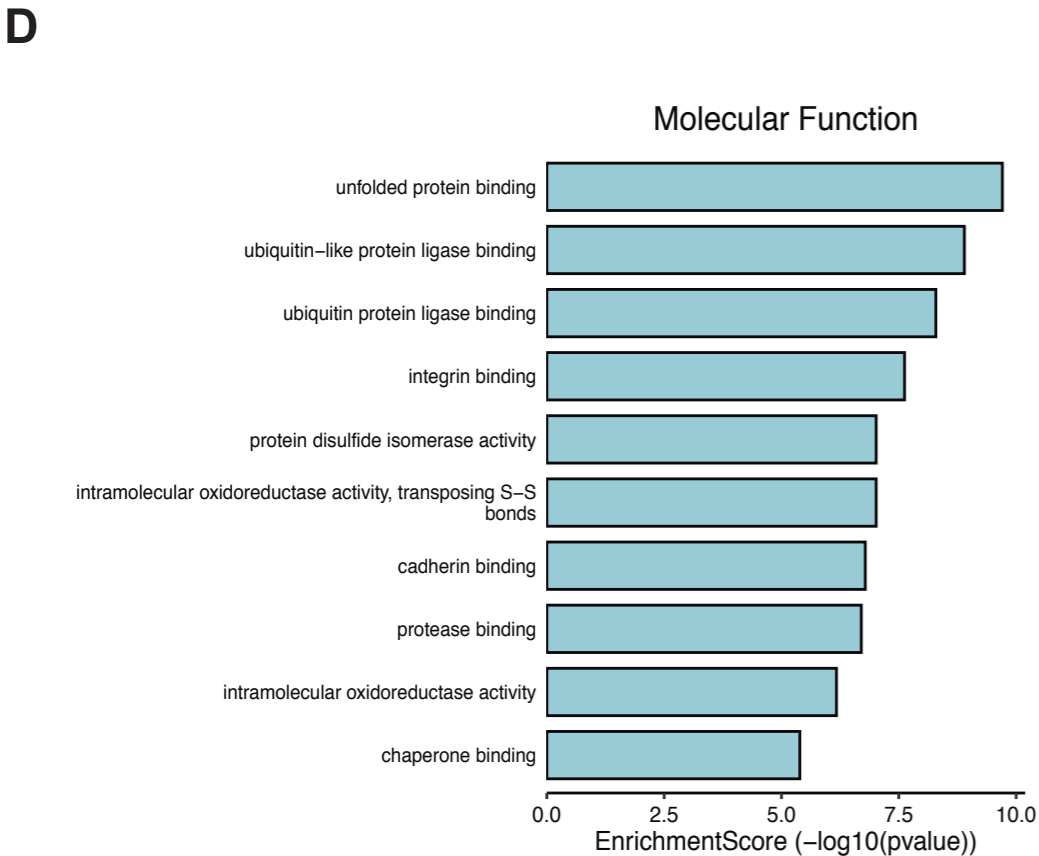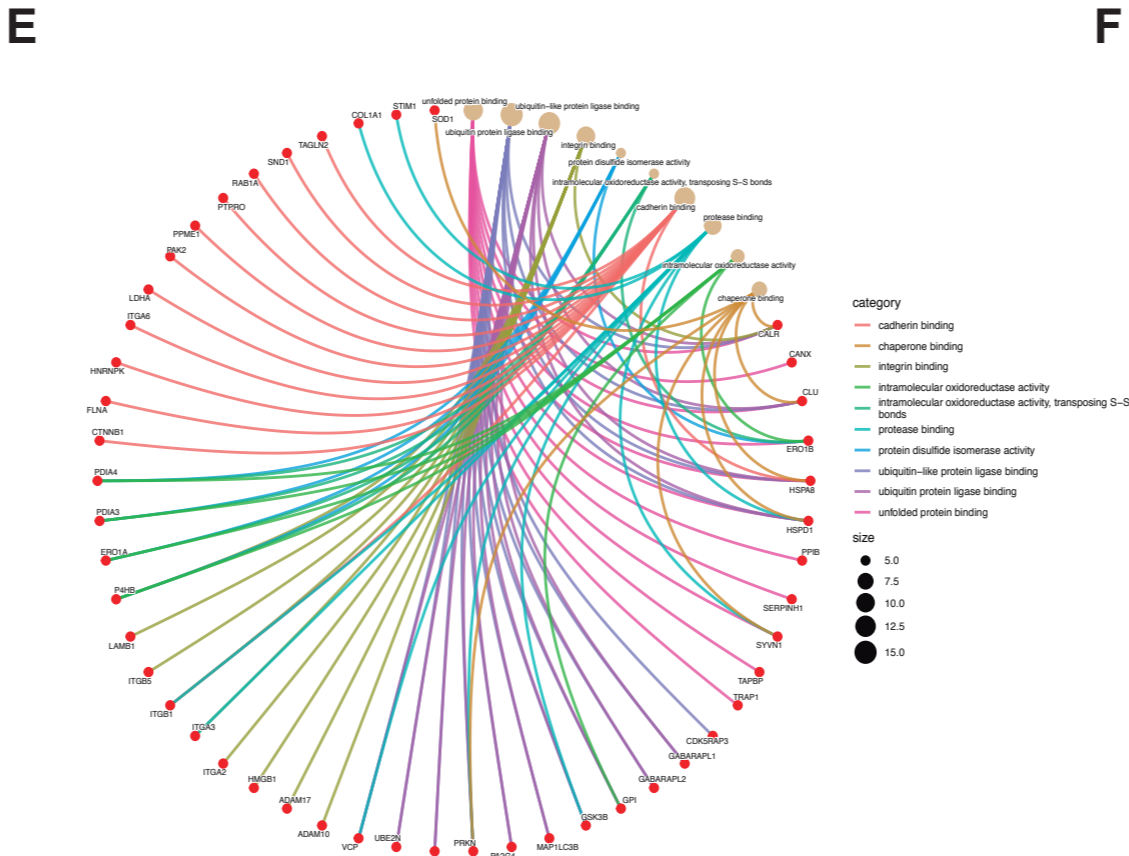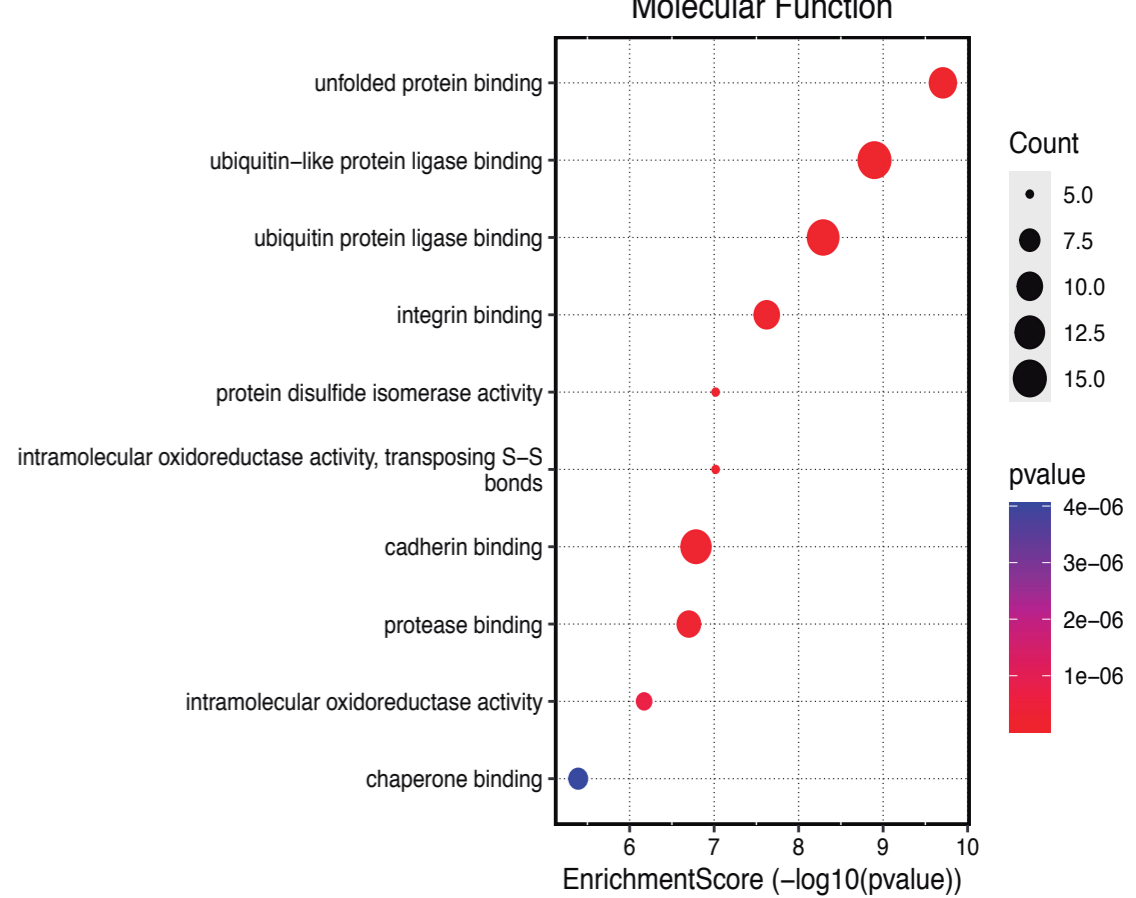

Supplement: Supplementary file 1 [file biomolecules-13-00848-s001.zip › Supplementary Figure S4.pdf]

A

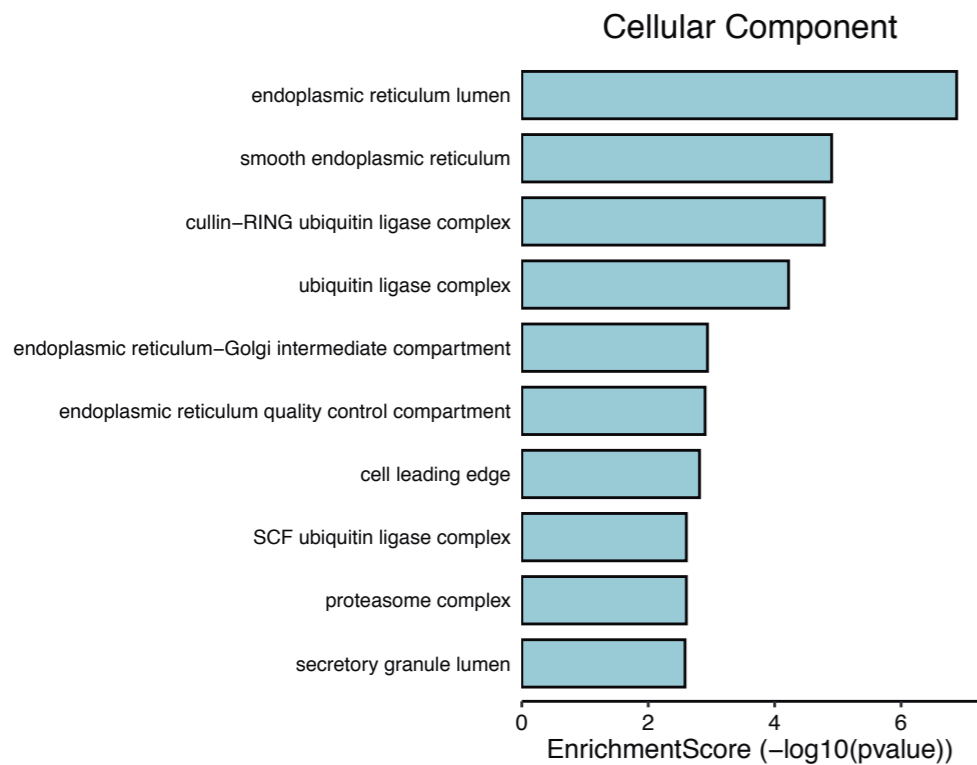

B

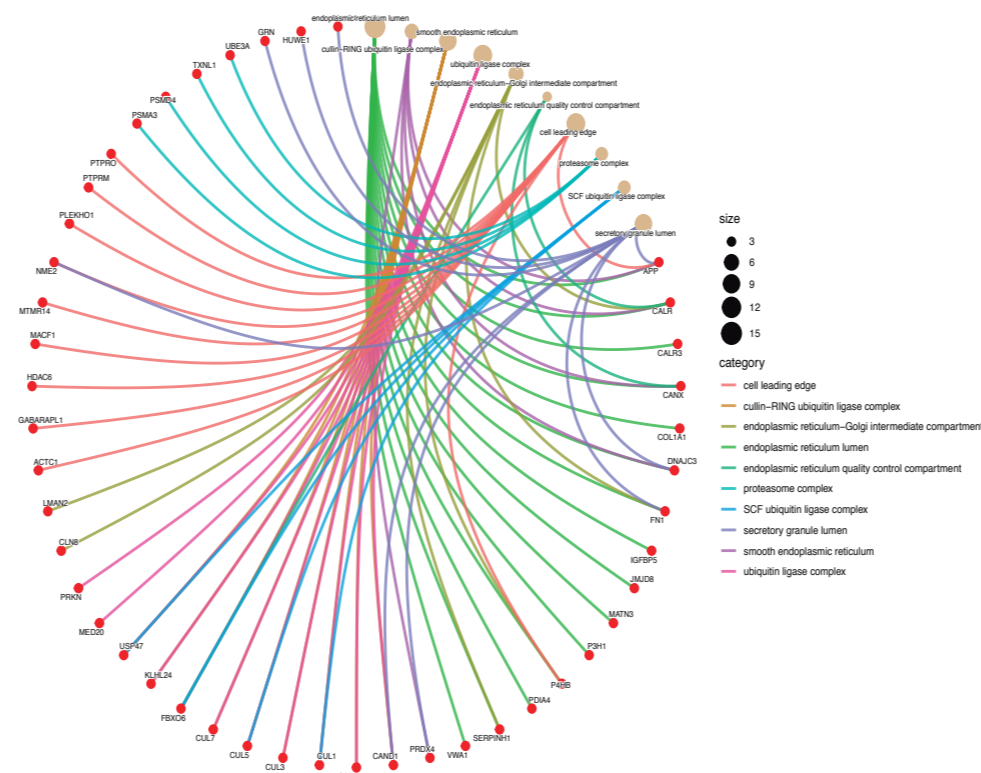

C

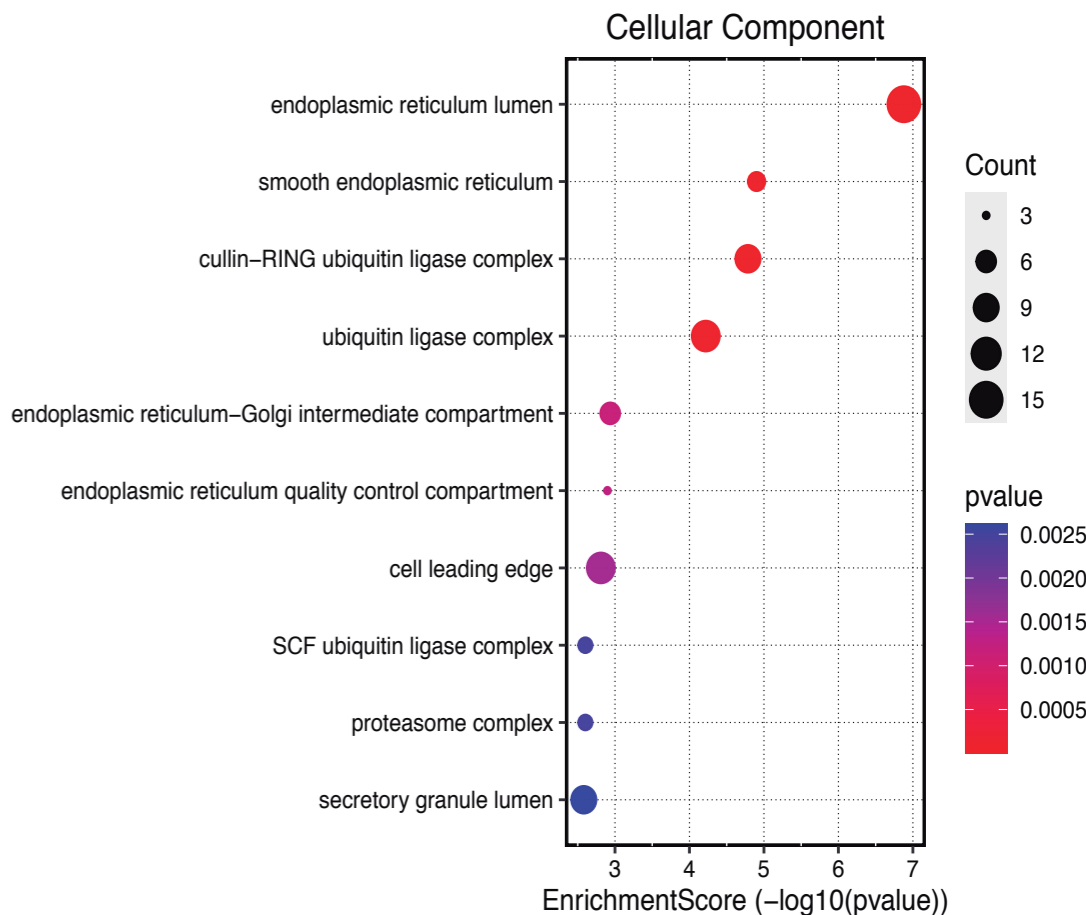

D

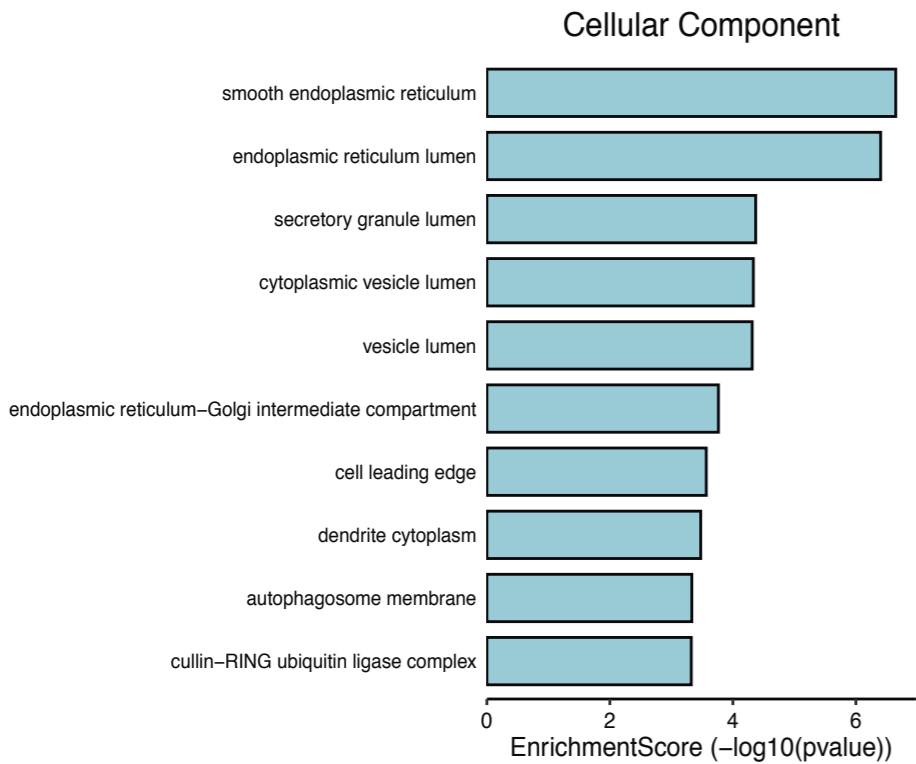

E

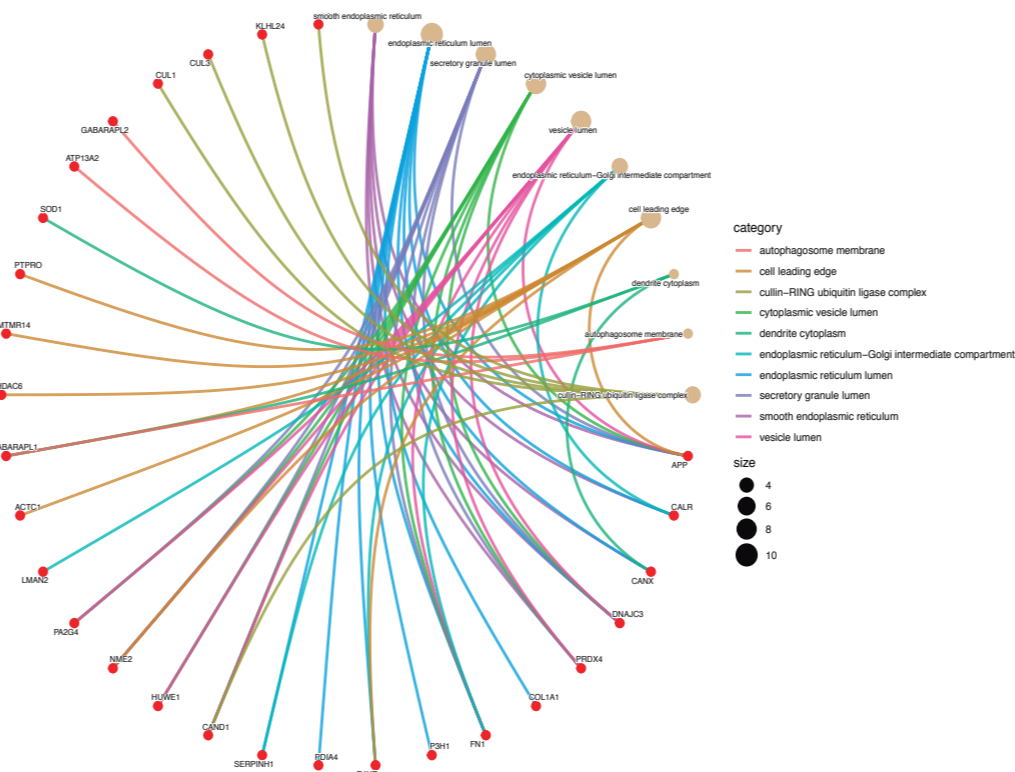

F

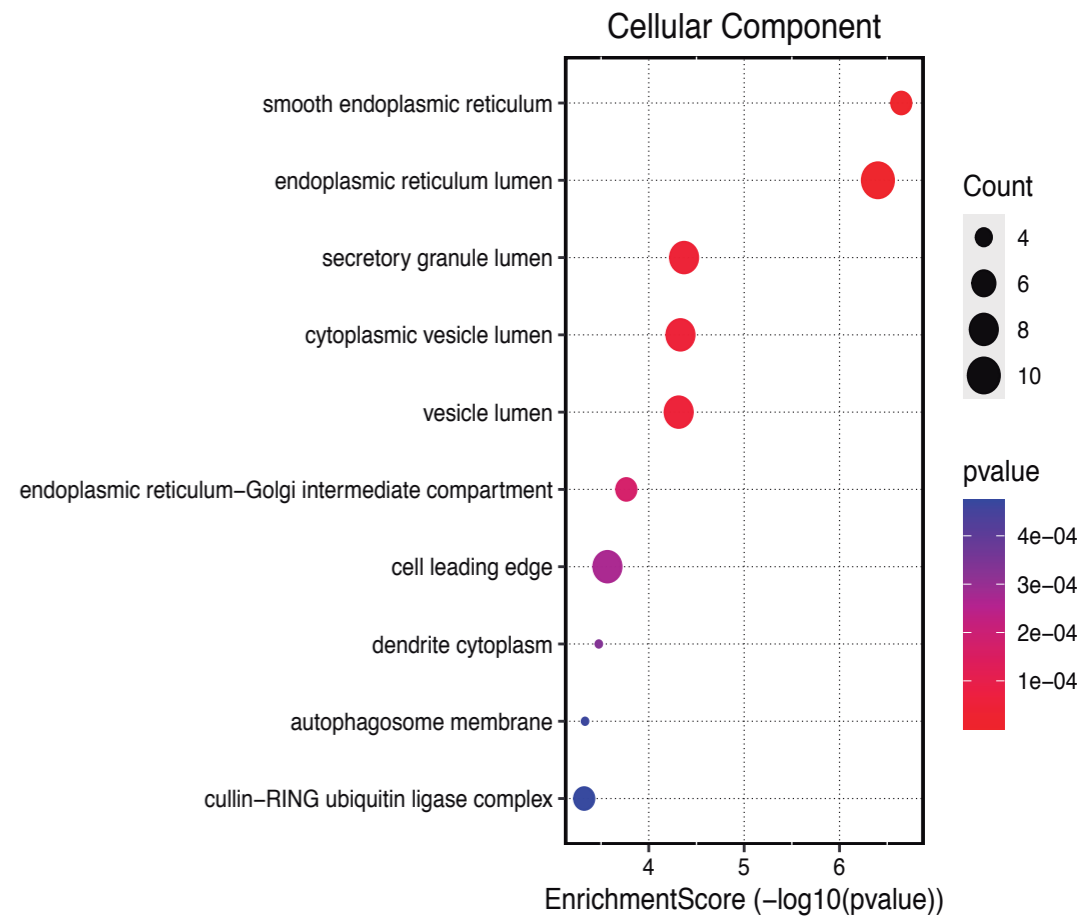

Supplement: Supplementary file 1 [file biomolecules-13-00848-s001.zip › Supplementary Figure S5.pdf]

PDIA6

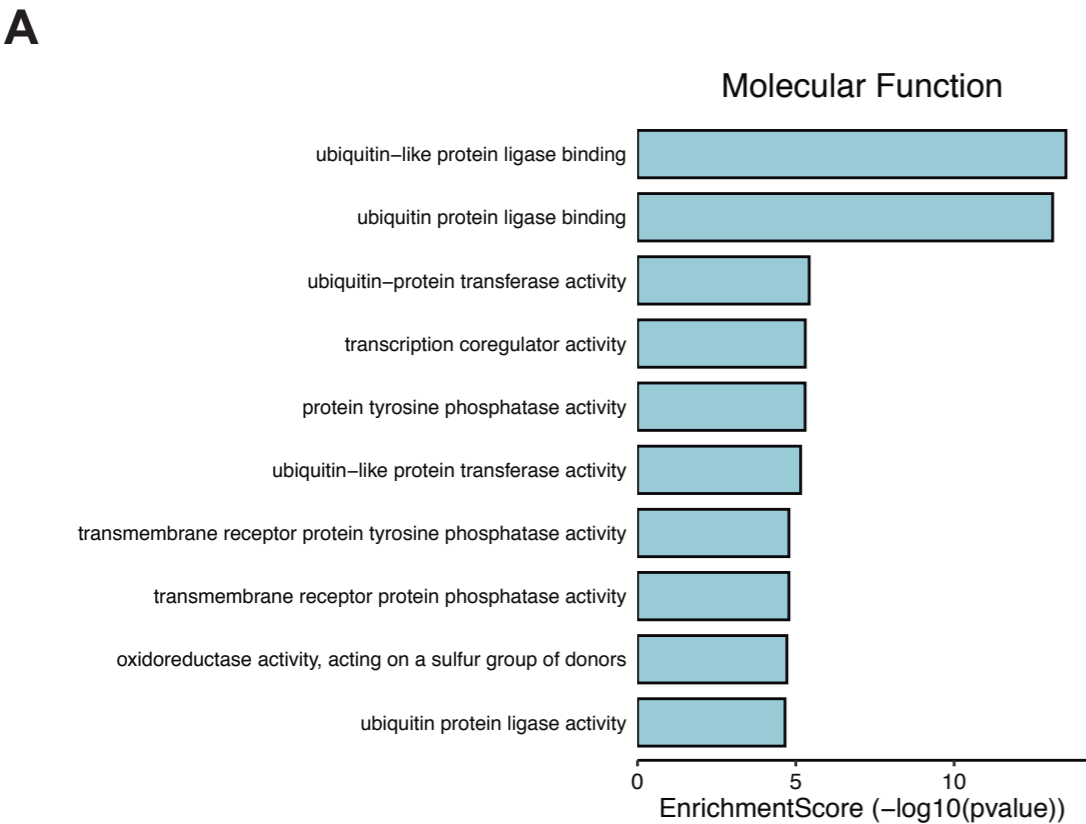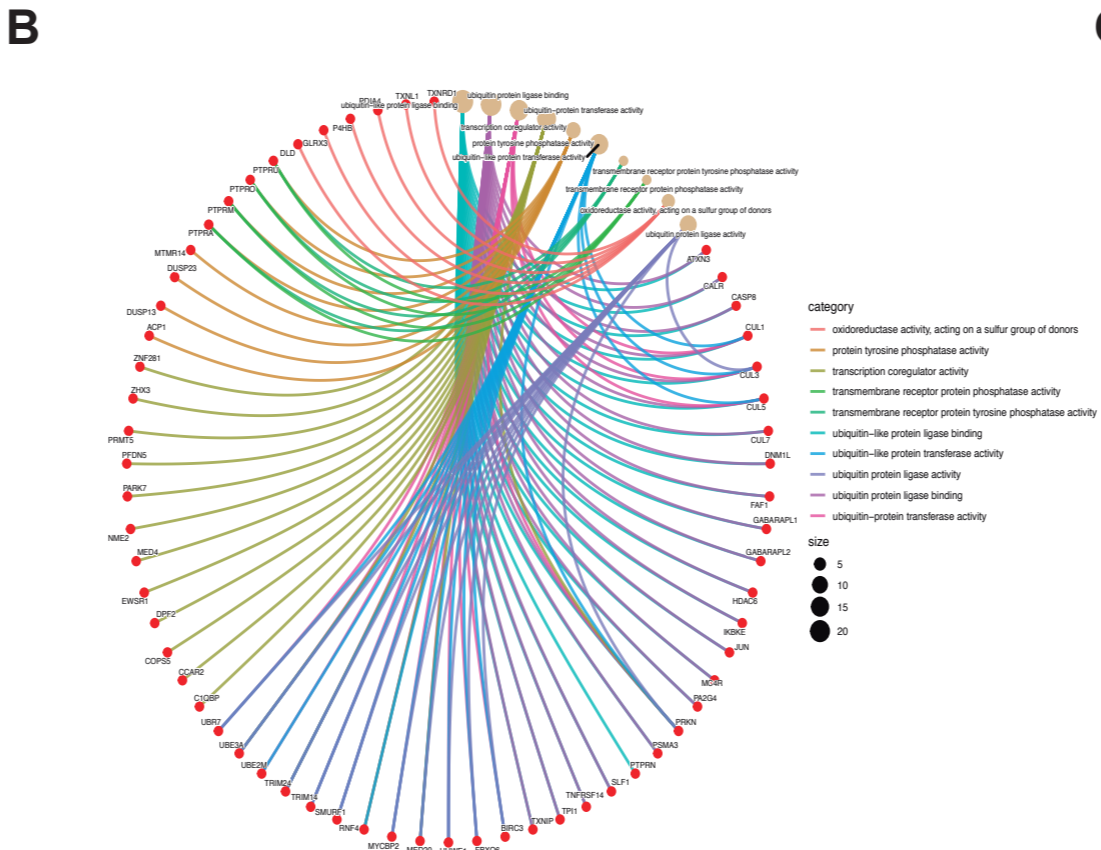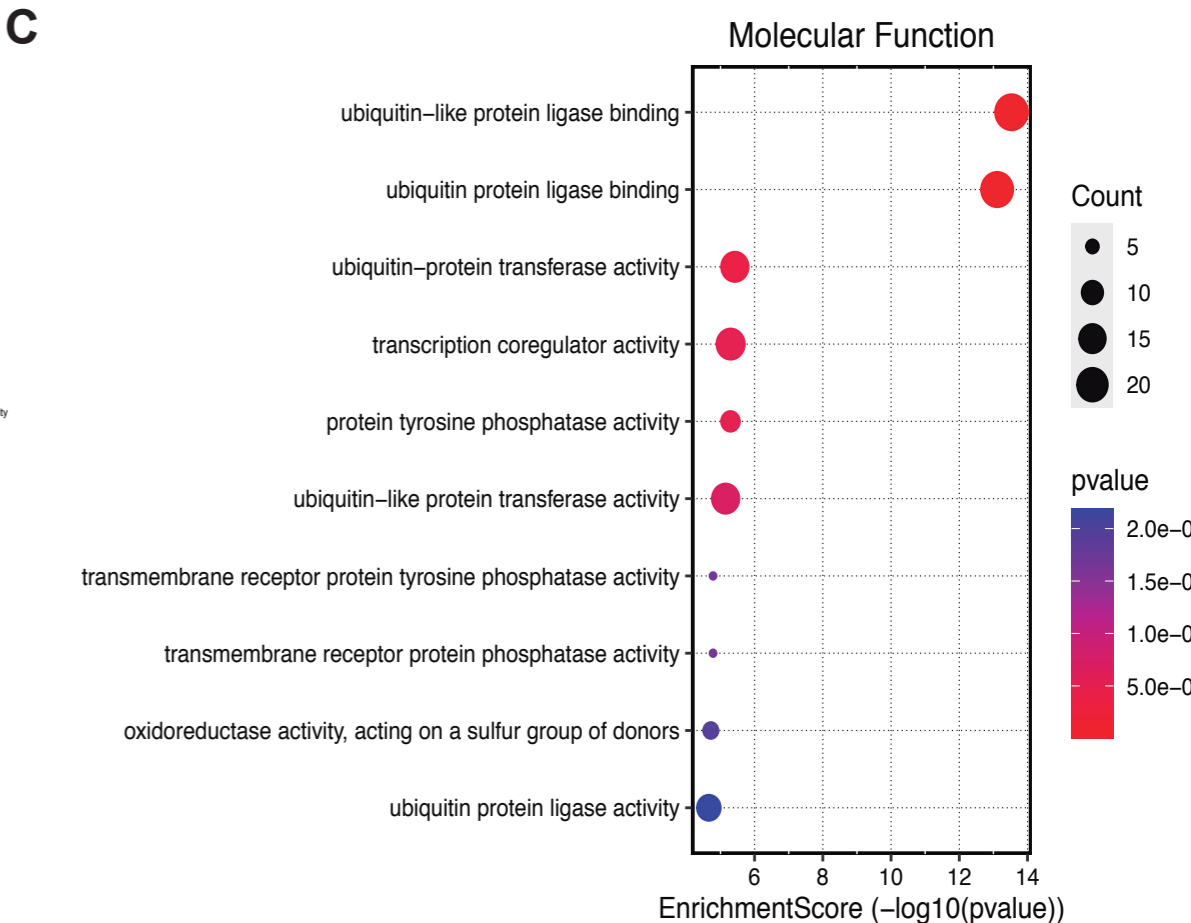

PDIA6-Platelets

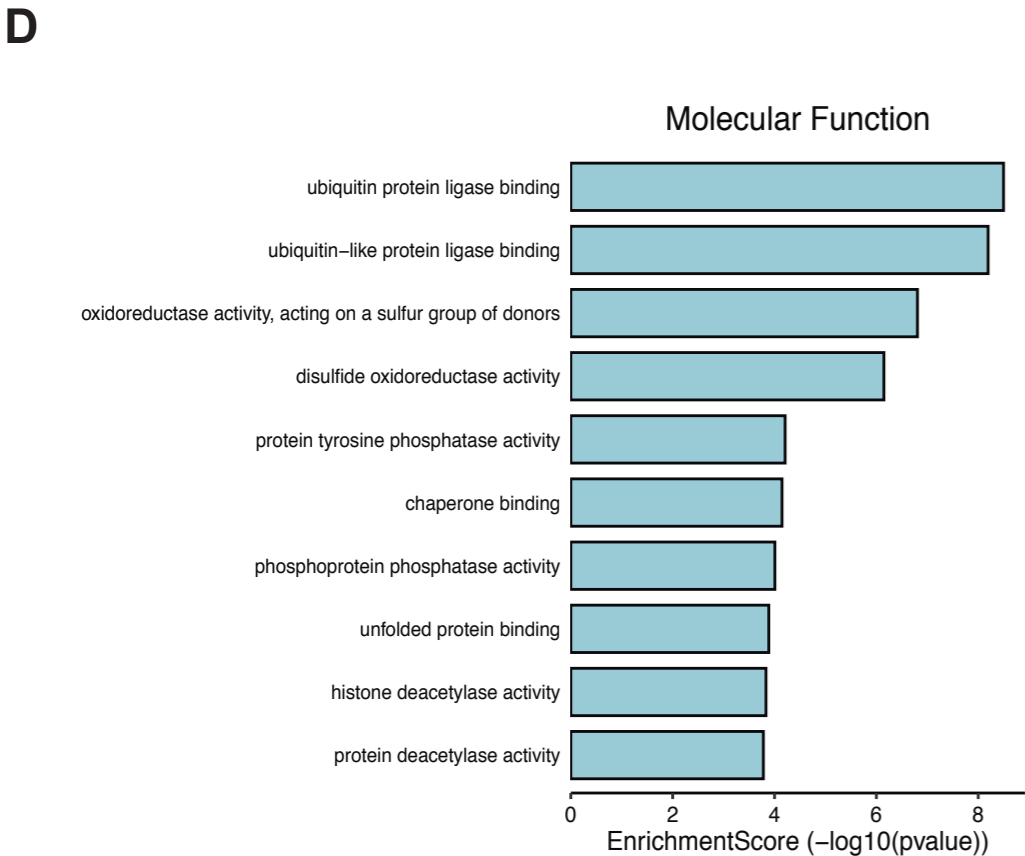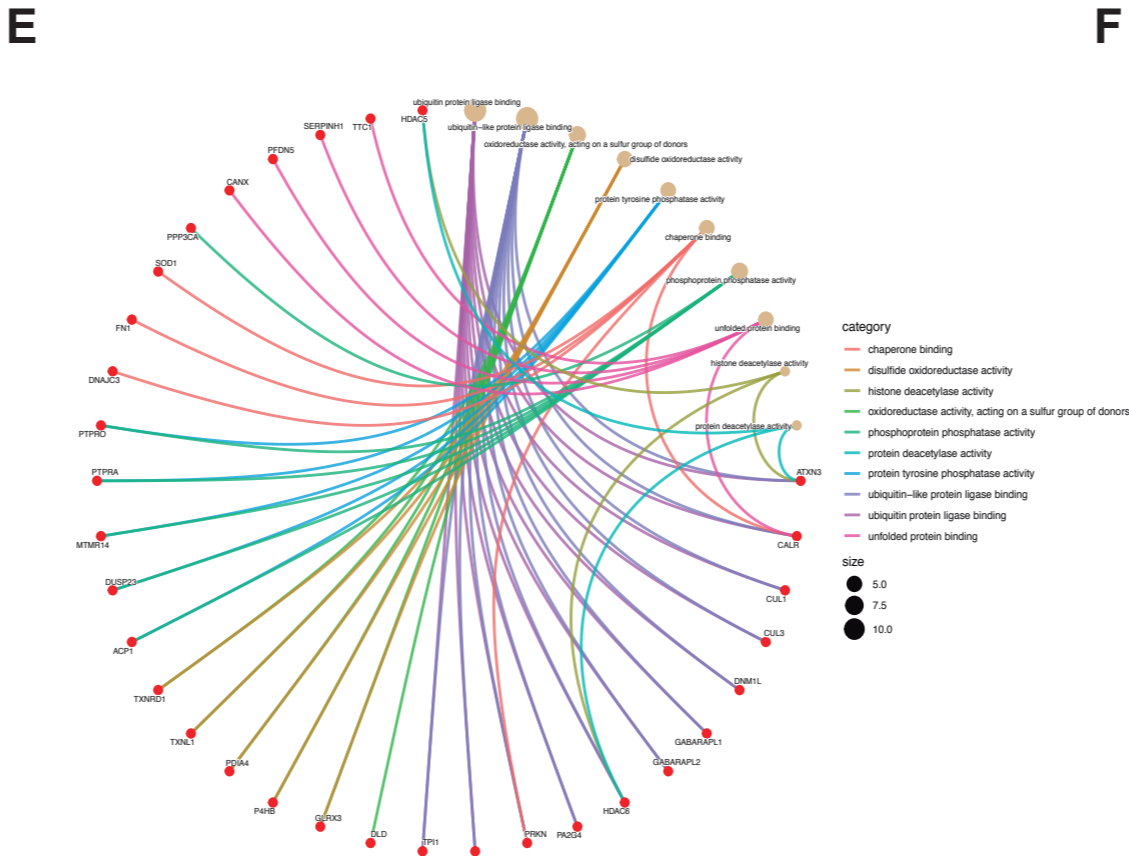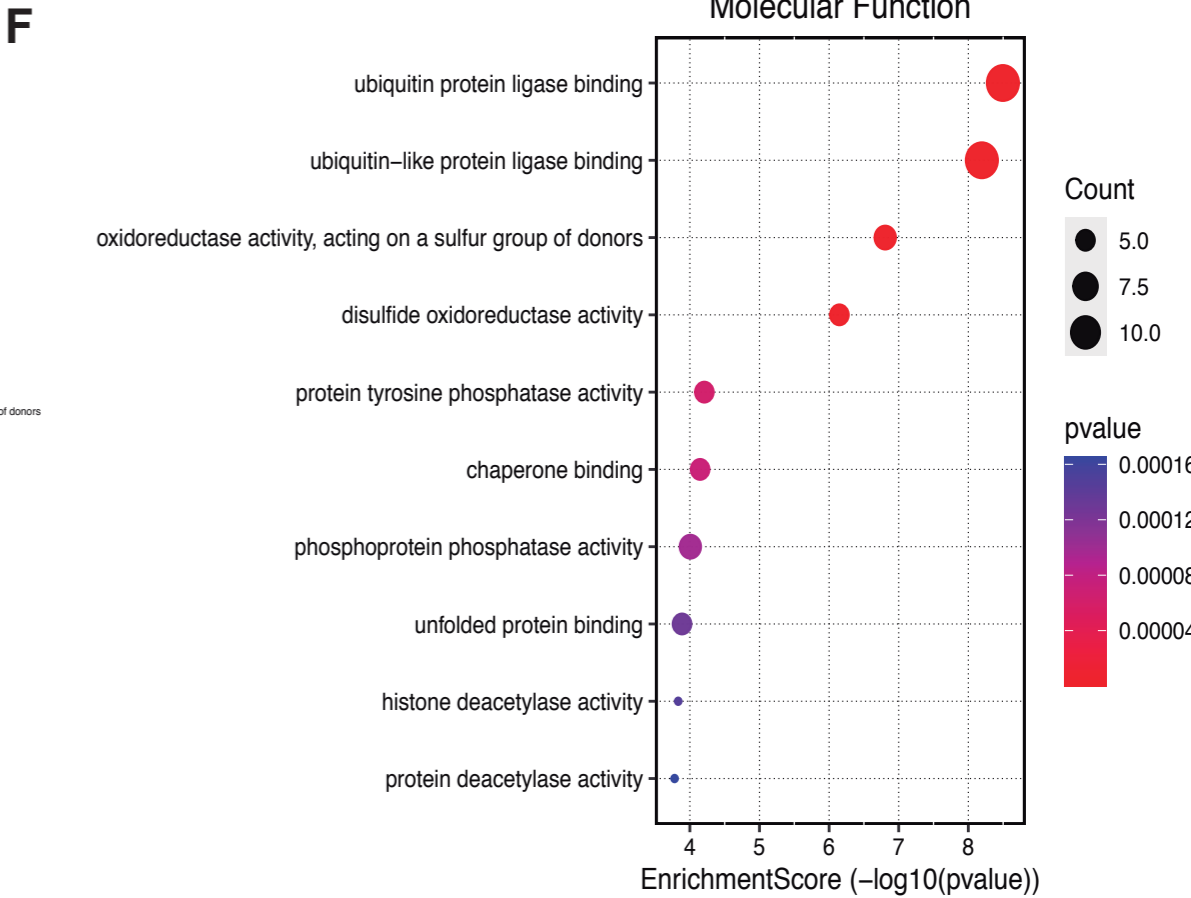

Supplement: Supplementary file 1 [file biomolecules-13-00848-s001.zip › Supplementary Figure S6.pdf]

A

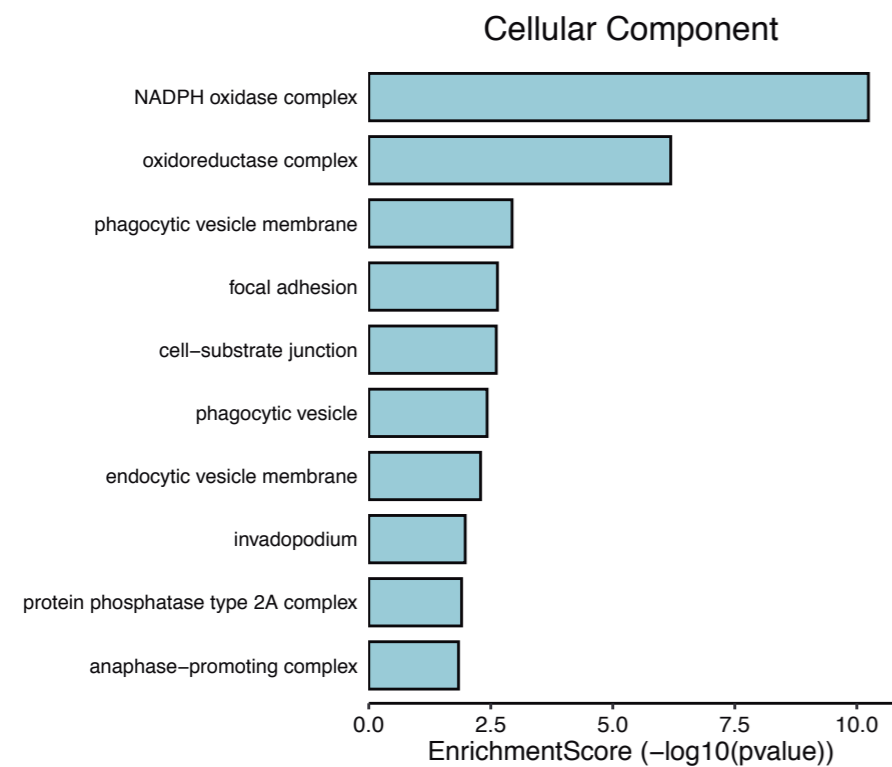

B

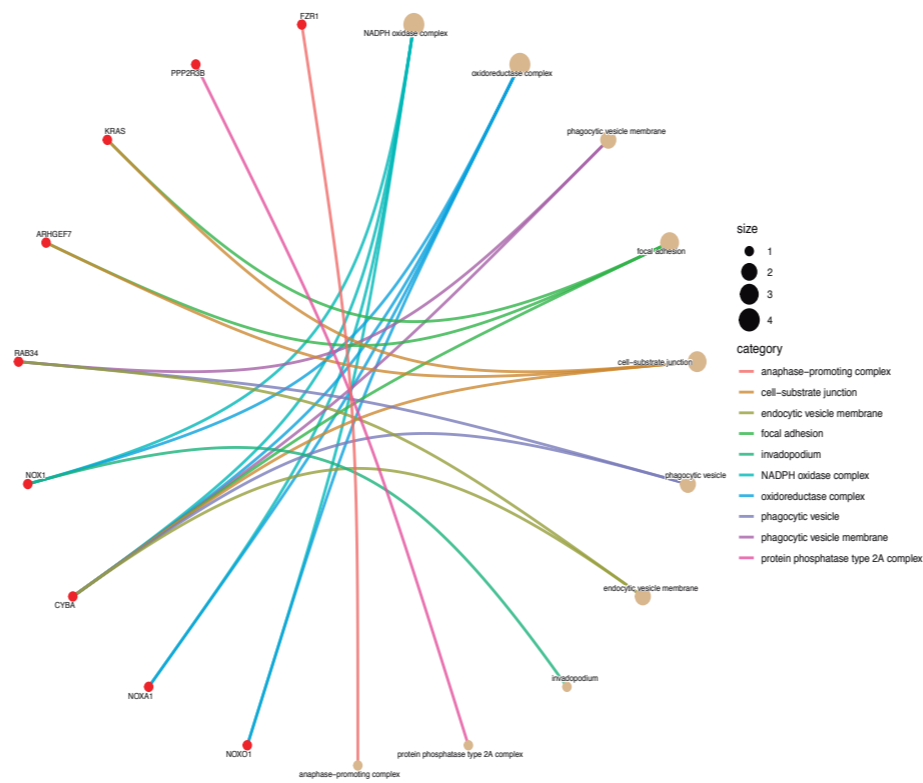

C

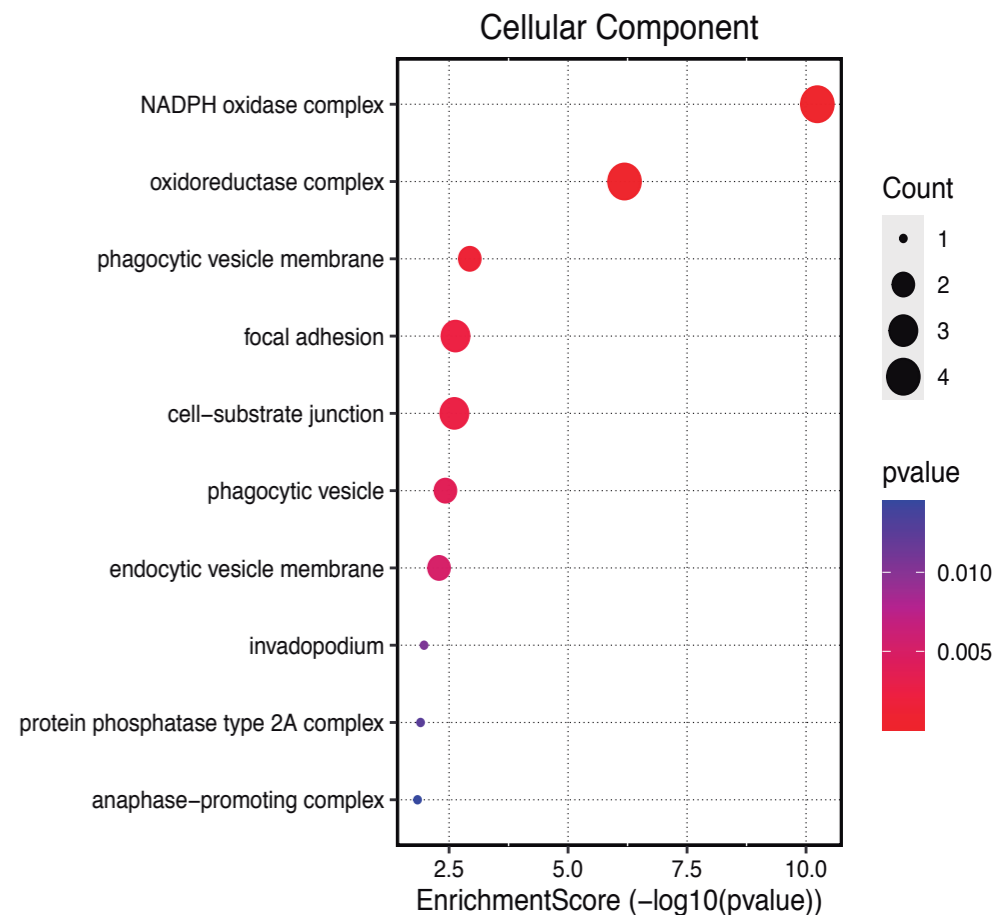

D

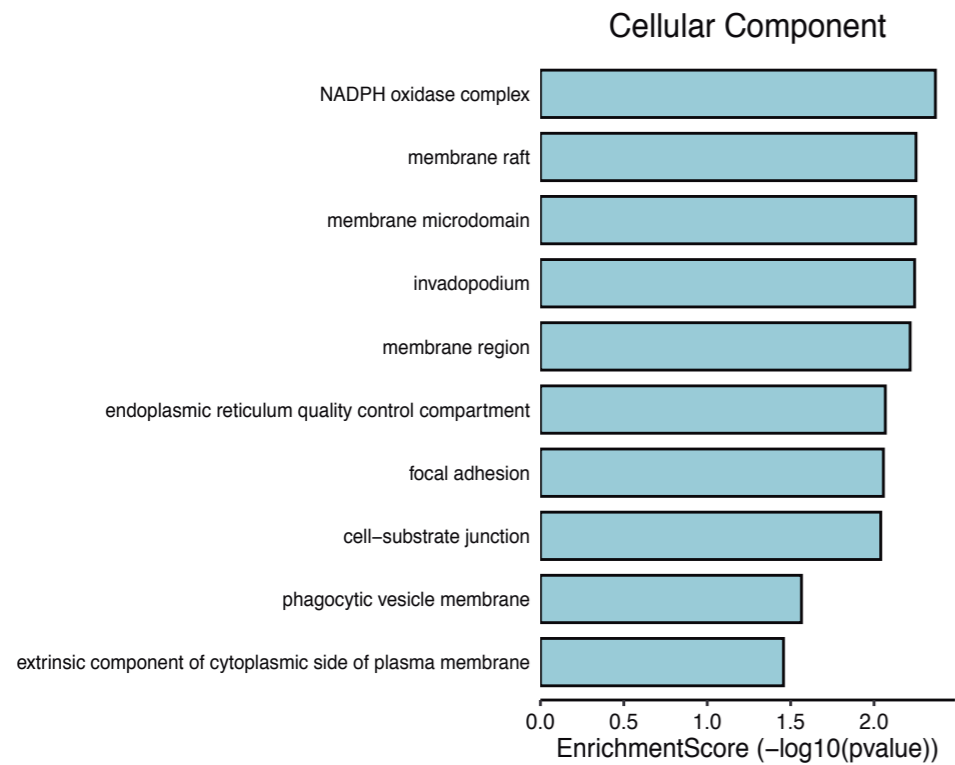

E

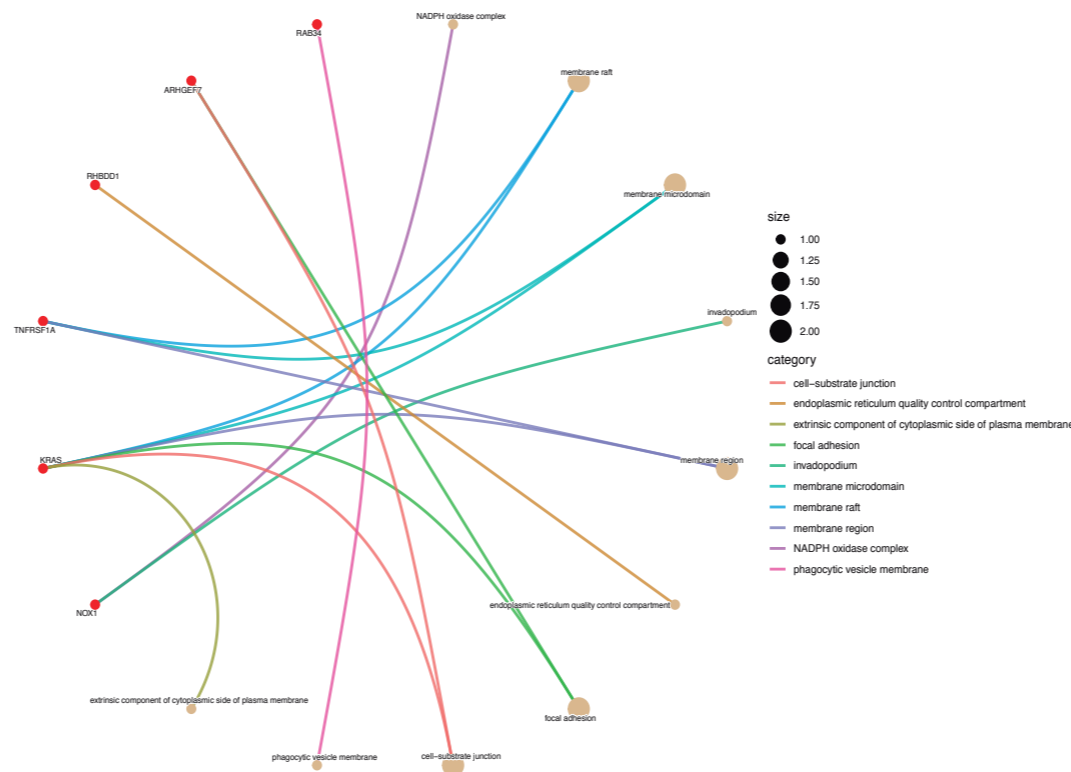

F

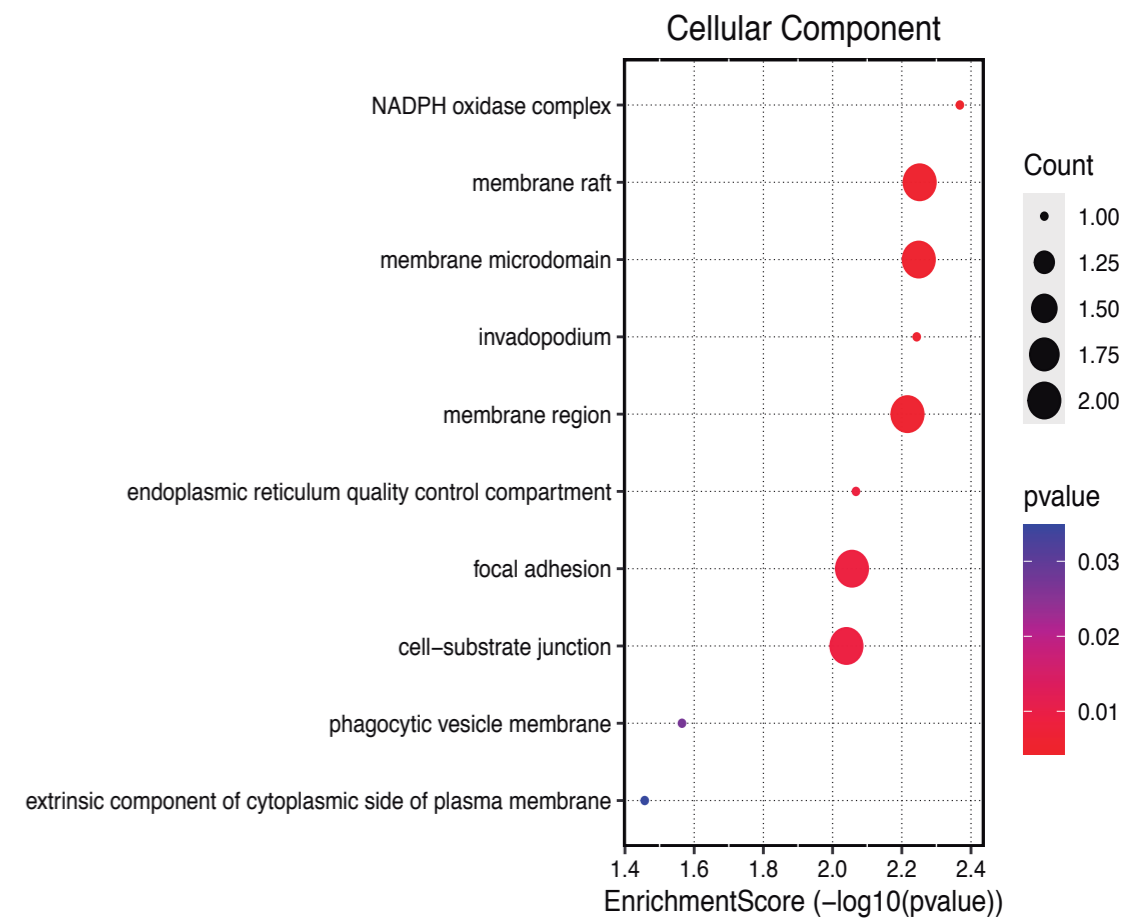

Supplement: Supplementary file 1 [file biomolecules-13-00848-s001.zip › Supplementary Figure S7.pdf]

NOX1

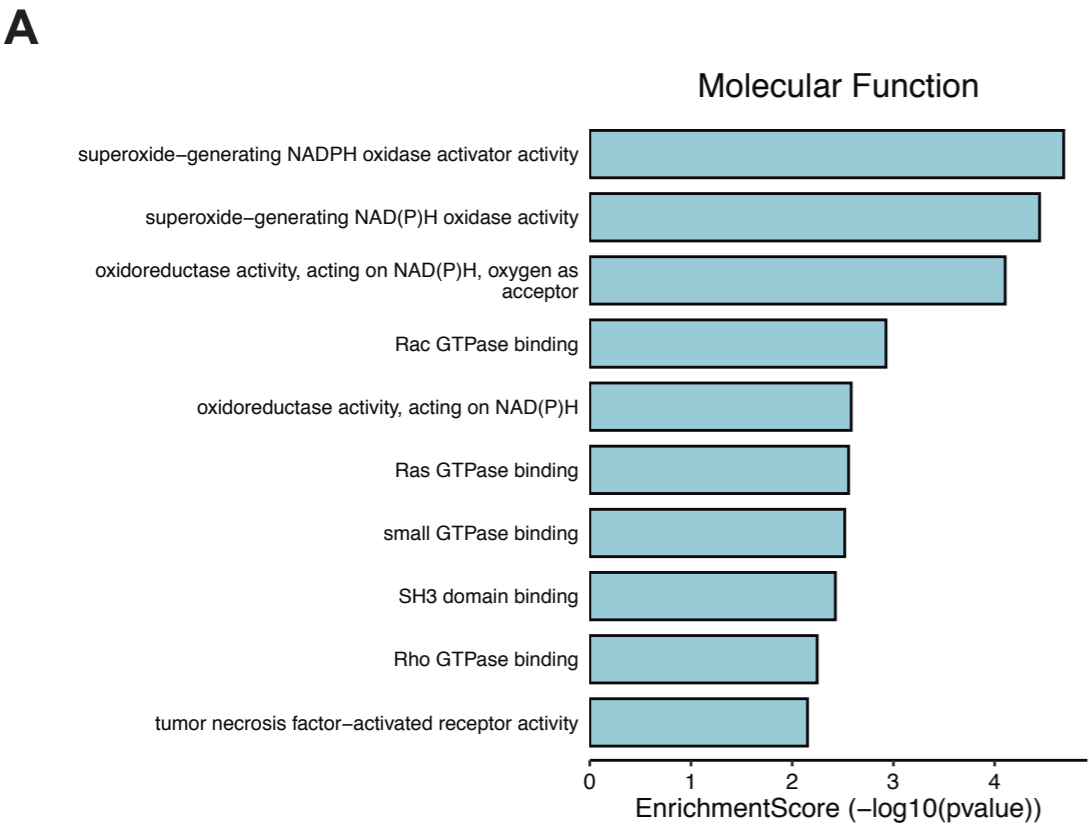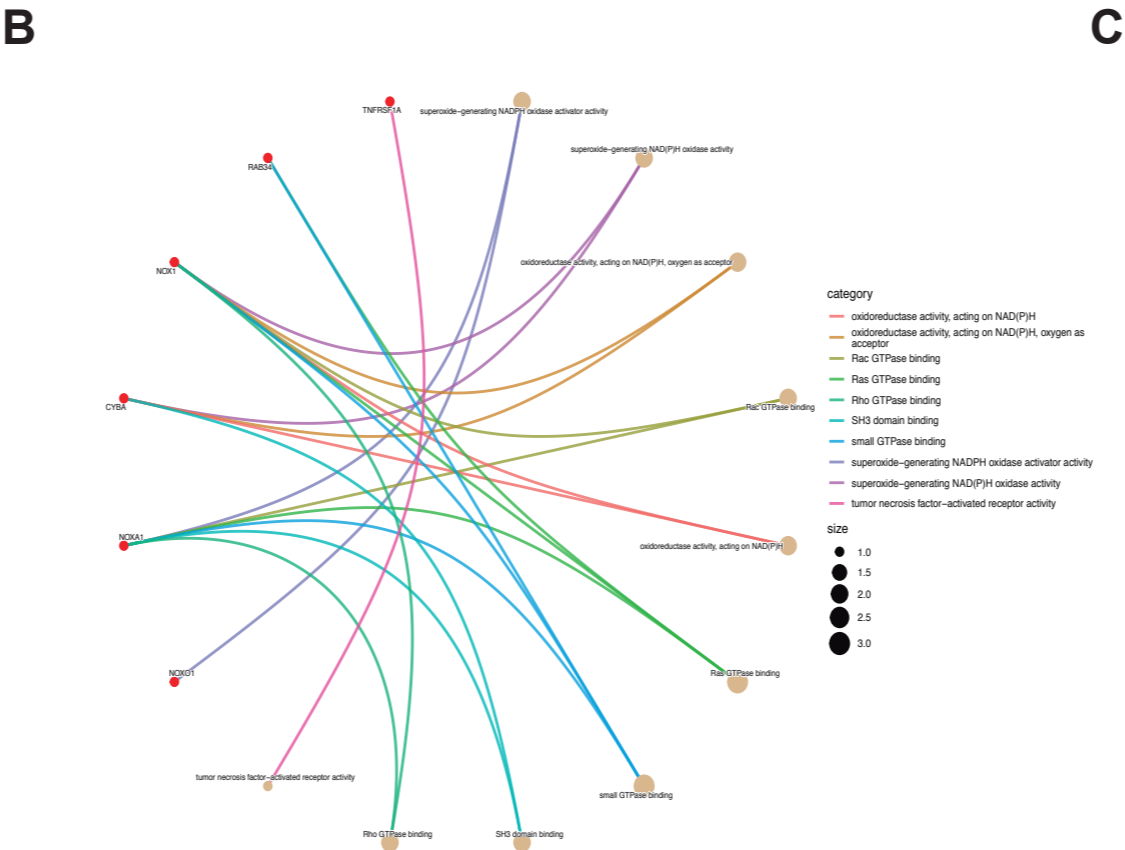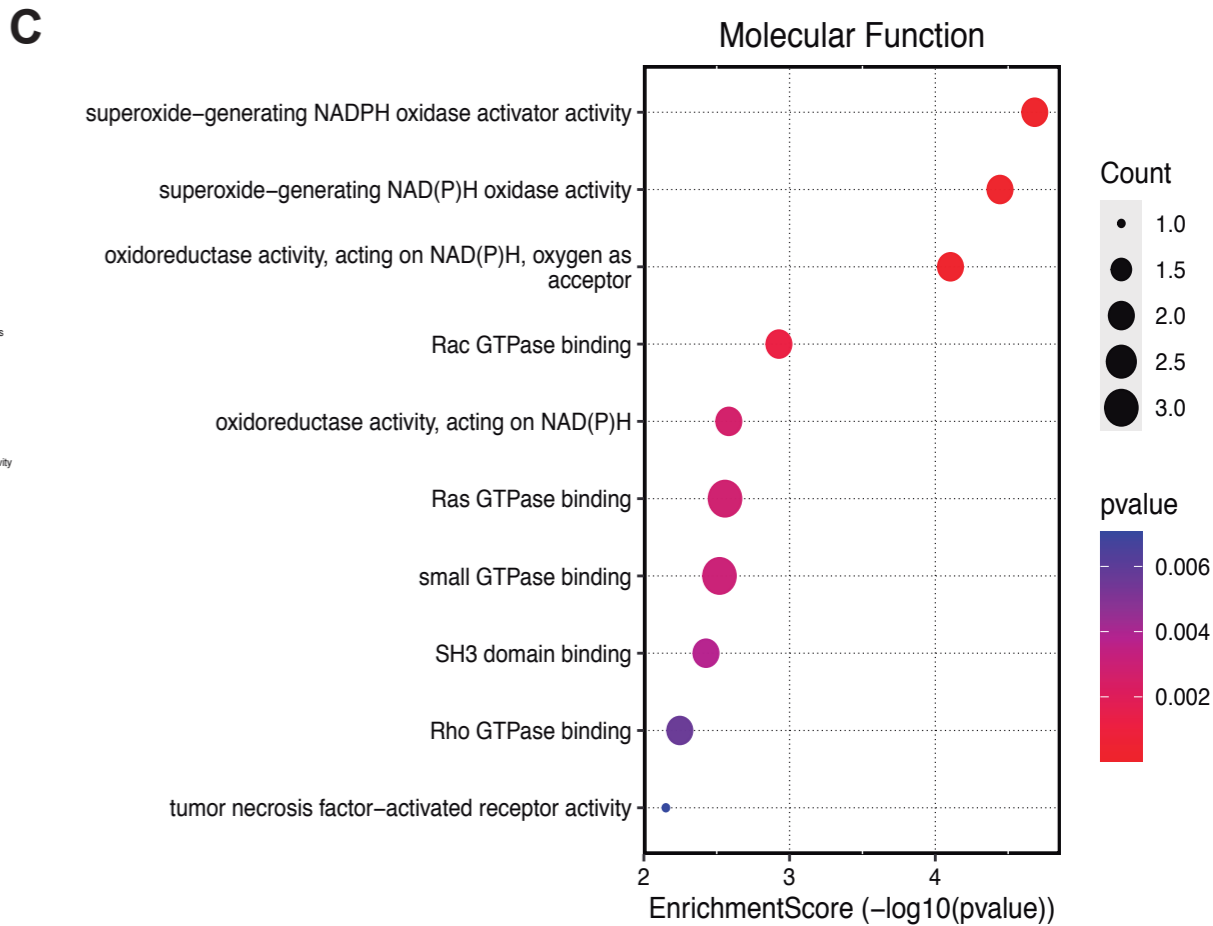

NOX1-Platelets

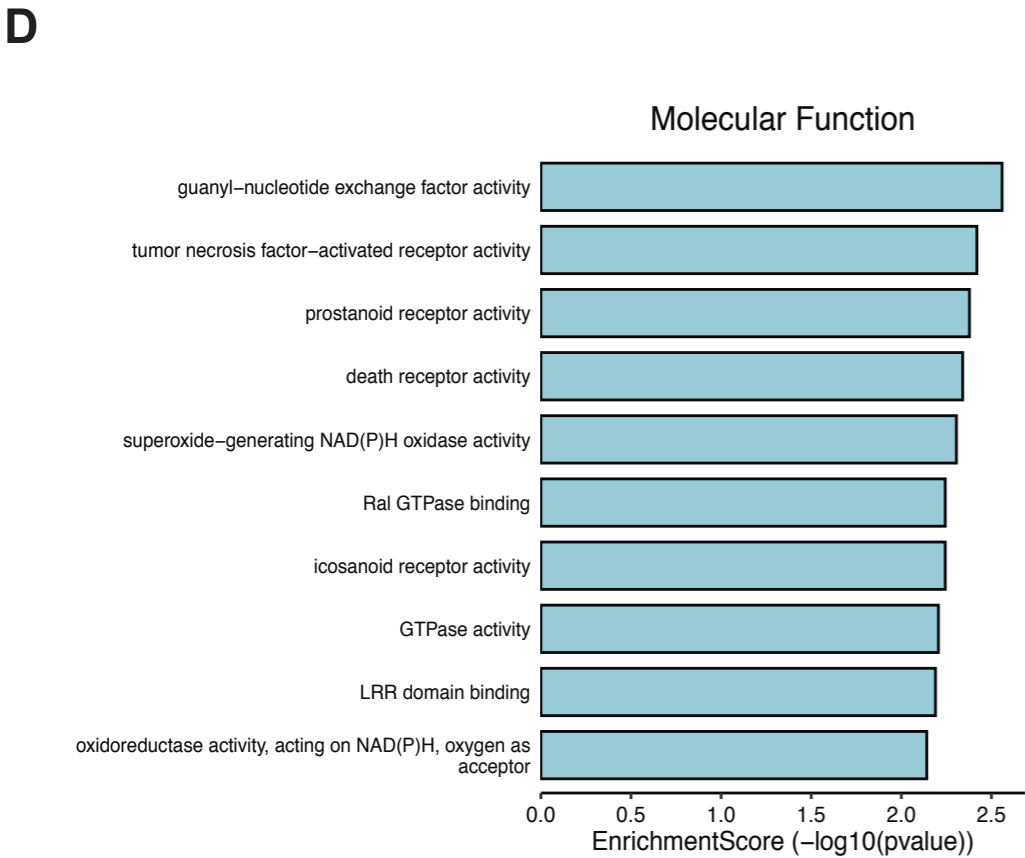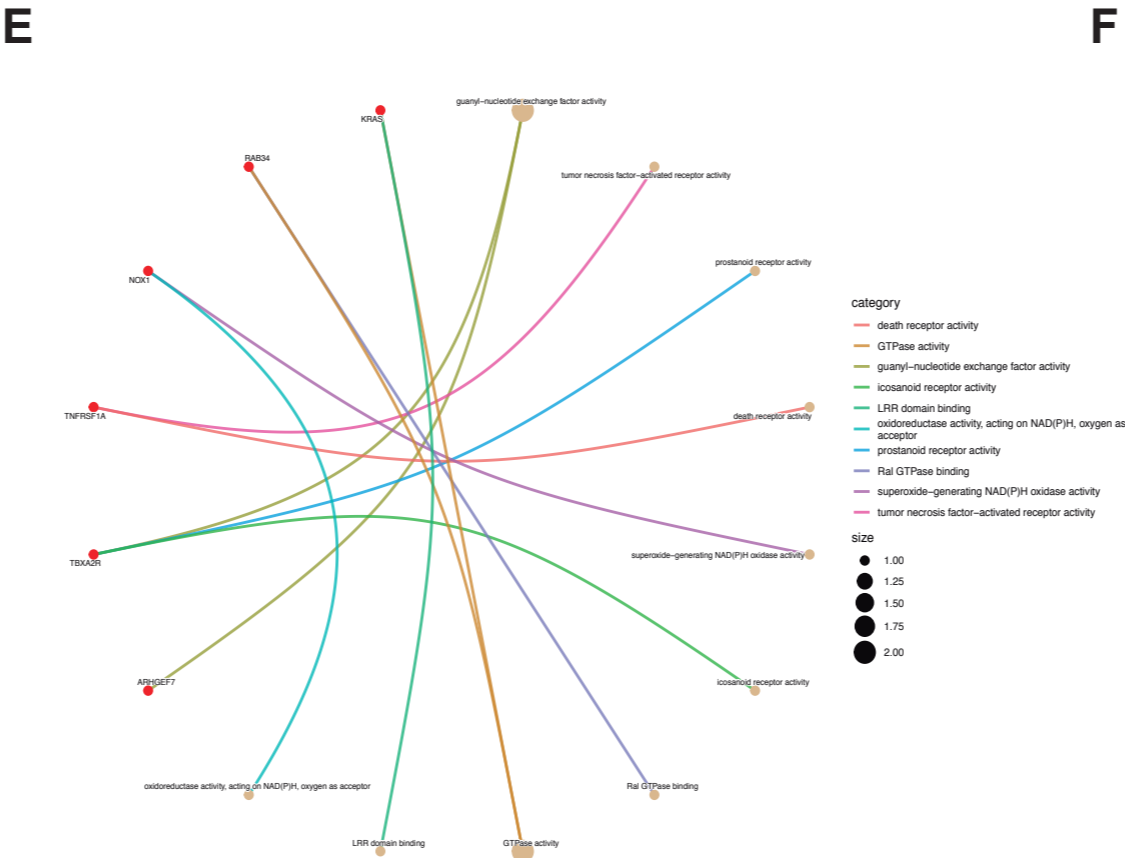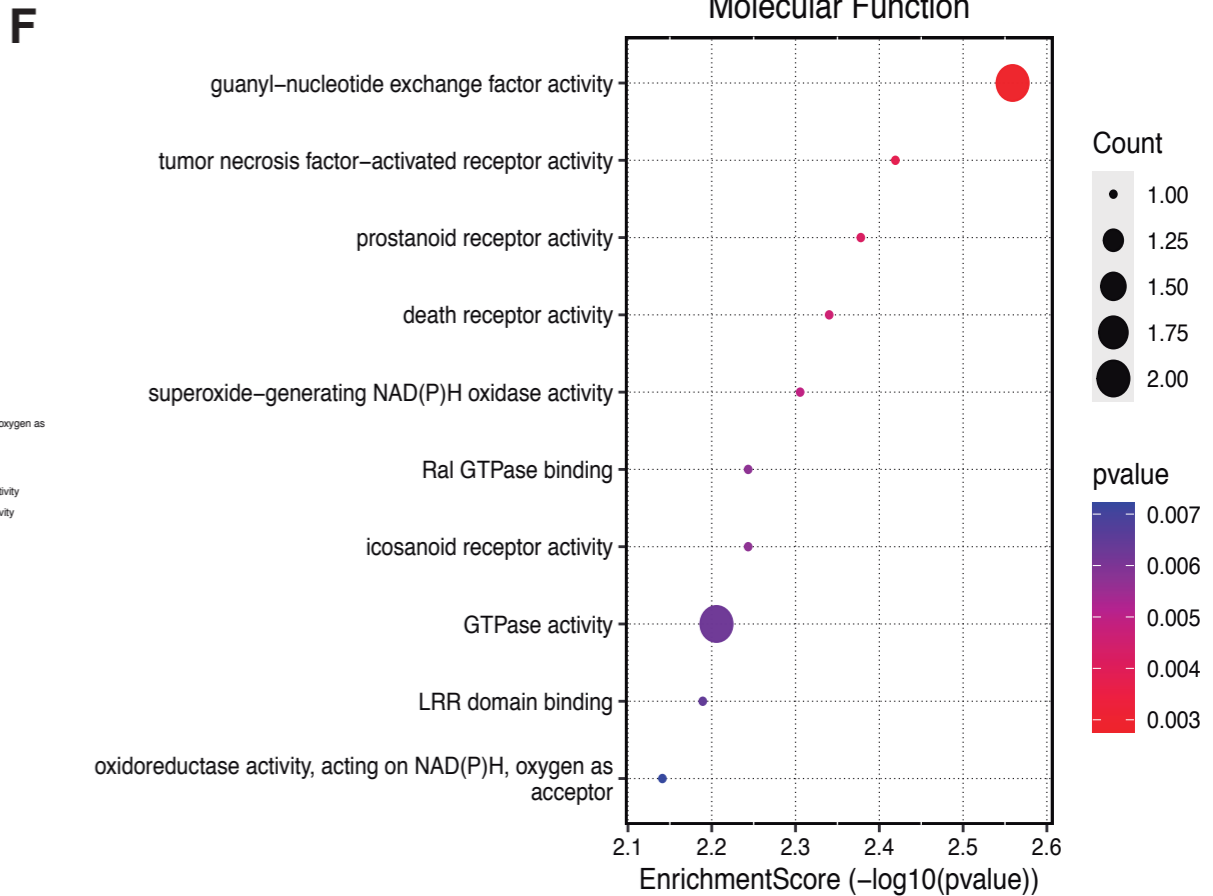

Supplement: Supplementary file 1 [file biomolecules-13-00848-s001.zip › Supplementary Figure S8.pdf]

A

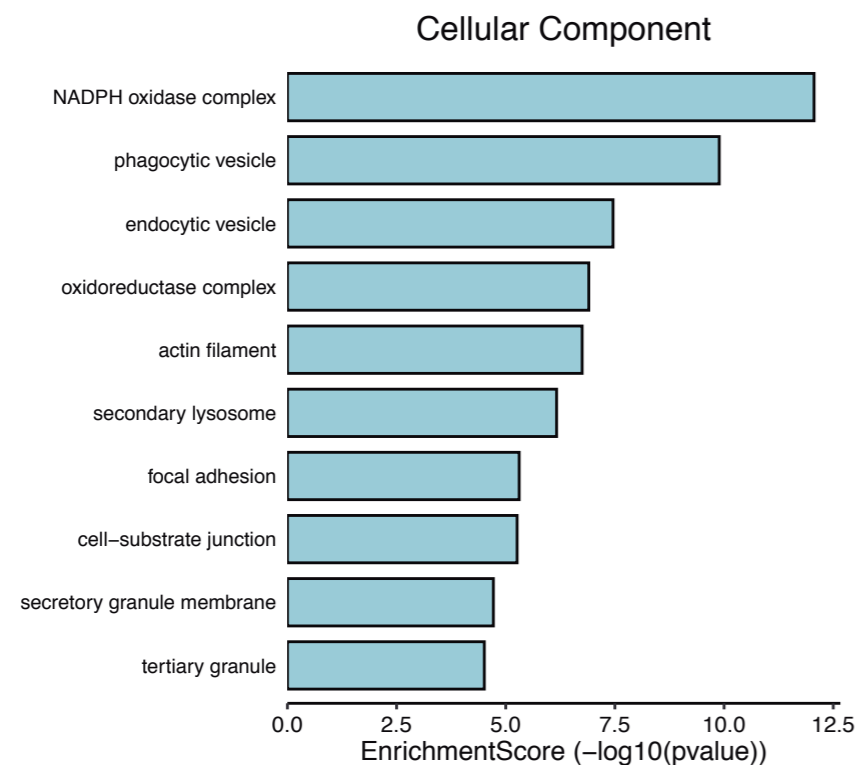

B

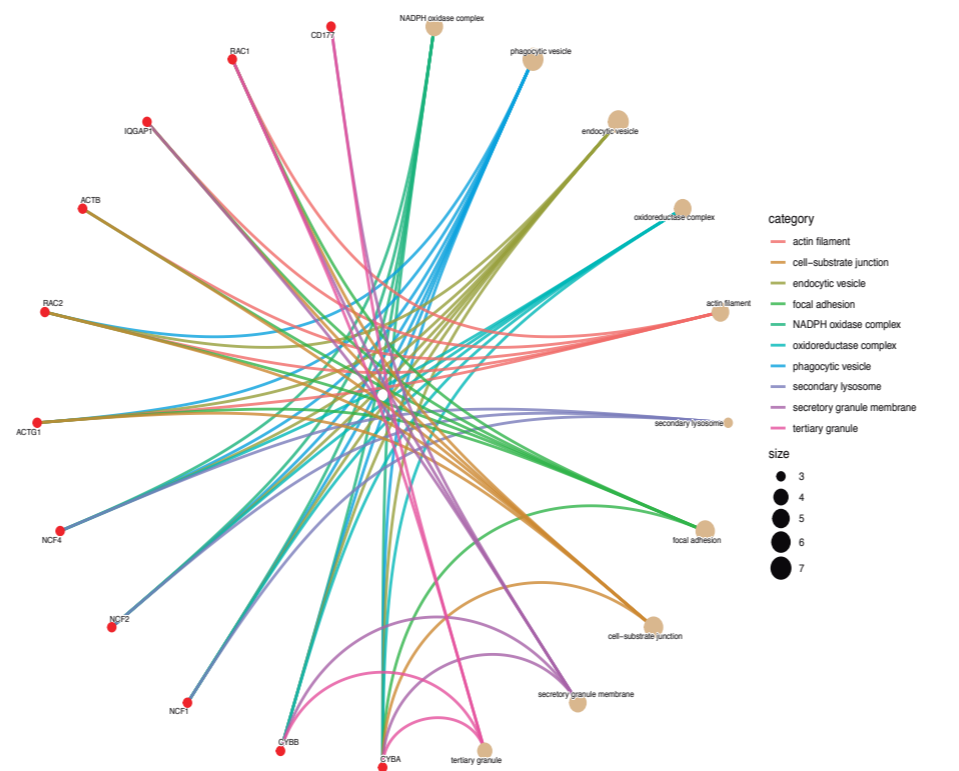

C

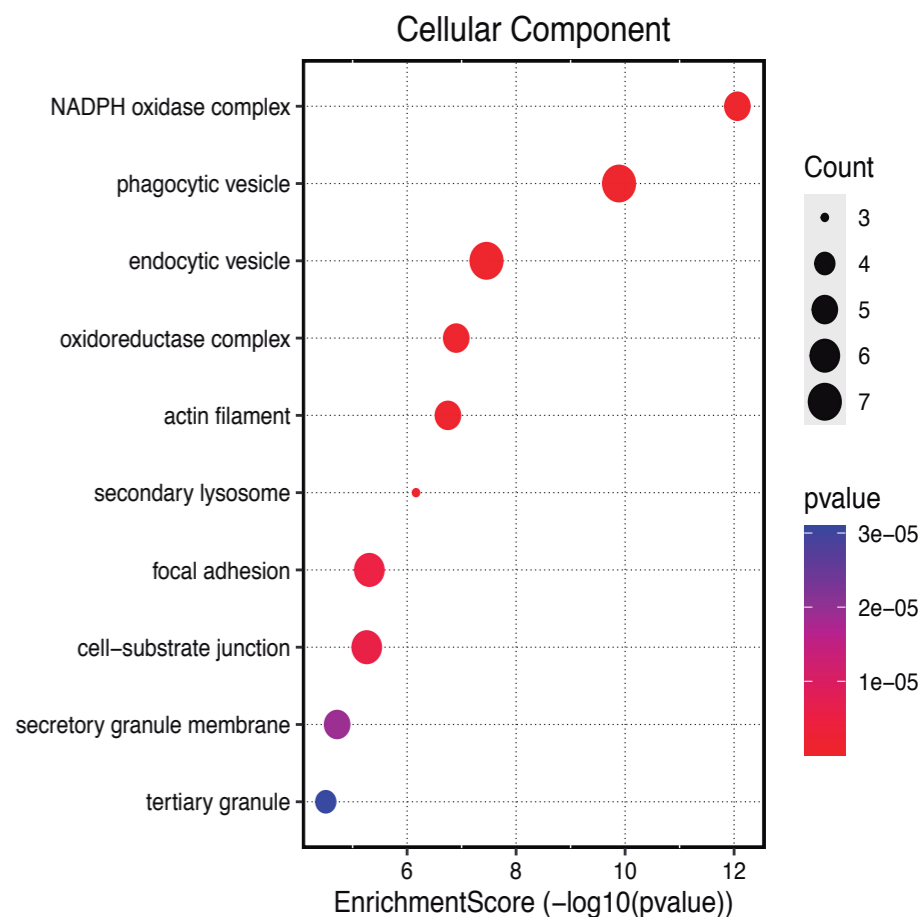

D

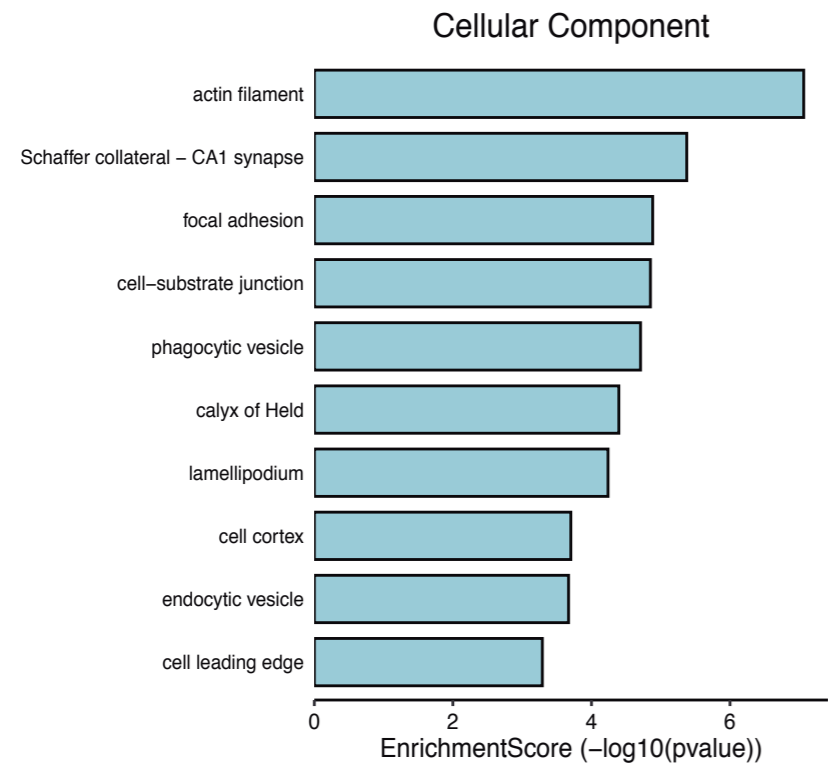

E

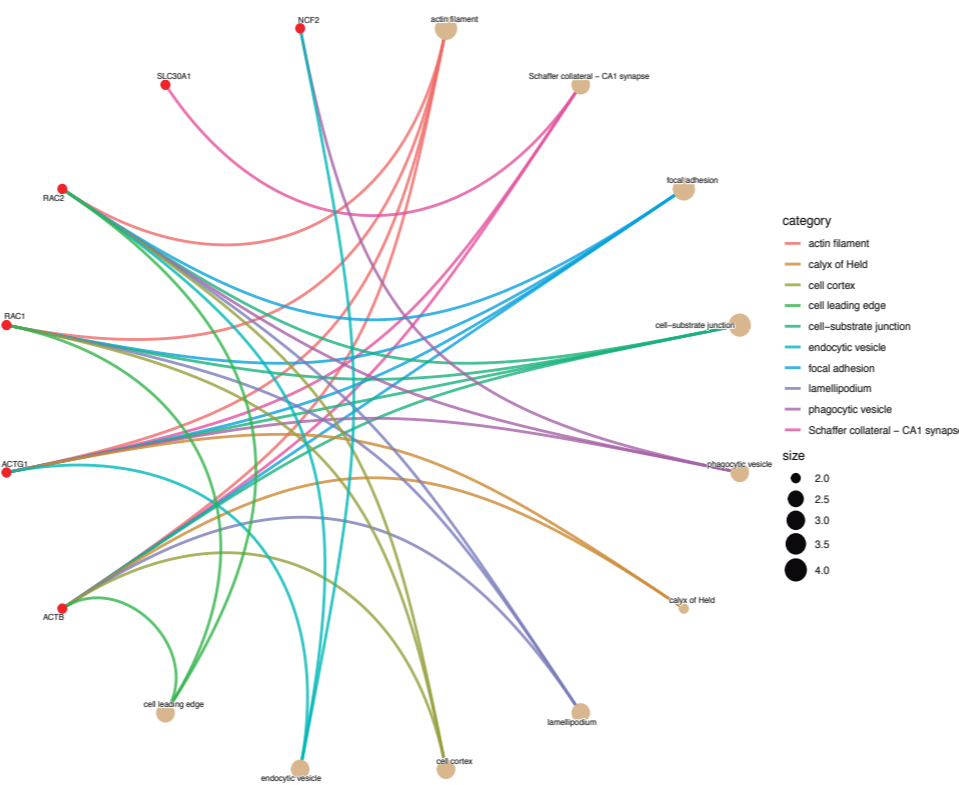

F

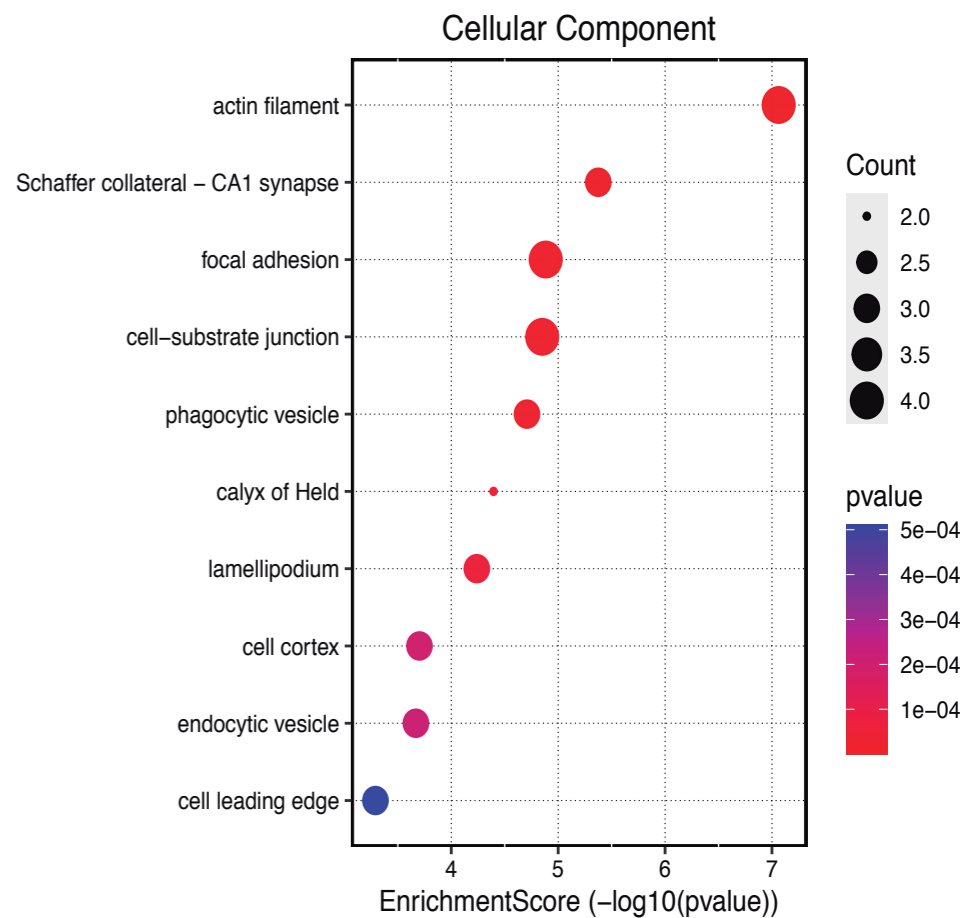

Supplement: Supplementary file 1 [file biomolecules-13-00848-s001.zip › Supplementary Figure S9.pdf]
